# Fatty Acid Synthase regulates glucose and energy homeostasis via POMC neurons and adrenergic signals

**DOI:** 10.1016/j.molmet.2025.102177

**Published:** 2025-06-18

**Authors:** Luis Leon-Mercado, Yanbin Dong, Bandy Chen, Arely Tinajero, Caleb C. Lord, Syann Lee, Chen Liu, Guosheng Liang, Jay D. Horton, Kevin W. Williams, Joel K. Elmquist

**Affiliations:** 1 Center for Hypothalamic Research, Department of Internal Medicine, University of Texas Southwestern Medical Center, Dallas, TX, USA; 2 Peter O'Donnell Jr. Brain Institute, University of Texas Southwestern Medical Center, Dallas, TX, USA; c Department of Molecular Genetics, University of Texas Southwestern Medical Center, Dallas, TX, USA

**Keywords:** FASN (Fatty Acid Synthase), POMC (Pro-Opiomelanocortin) neurons, Energy balance, Glucose metabolism, Adrenergic signaling

## Abstract

**Objectives:**

Hypothalamic Fatty Acid Synthase (FASN) plays a critical role in regulating energy balance by influencing food intake and body weight. This study aimed to investigate the neuronal mechanisms by which FASN impacts metabolism, focusing on its role in Pro-Opiomelanocortin (POMC) neurons.

**Methods:**

We used transgenic mouse models with pre- or postnatal deletion of FASN specifically in POMC neurons in male mice. We evaluated changes in adiposity, glucose metabolism and metabolic parameters including food intake, energy expenditure and substrate utilization using metabolic chambers. Changes in neuronal activity were assessed using electrophysiology and further validated by optogenetic stimulation of POMC neurons. Additionally, the role of adrenergic signaling was examined using pharmacological approaches and gene expression analyses.

**Results:**

FASN deletion in POMC neurons reduced food intake, decreased adiposity, and altered glucose metabolism. FASN-deficient POMC neurons exhibited increased baseline activity. The developmental stage of FASN deletion influenced its effects on energy expenditure and body weight regulation. Additionally, FASN in POMC neurons was found to be essential for maintaining glucose homeostasis and insulin release via adrenergic signaling.

**Conclusions:**

FASN in POMC neurons plays an age- and neuron-specific role in regulating feeding, energy expenditure, and glucose homeostasis through mechanisms including the sympathetic nervous system. These findings highlight FASN as a potential therapeutic target for metabolic diseases by improving energy expenditure and insulinemia. Given the developmental programming of metabolic outcomes, interventions aimed at modulating FASN activity may have long-lasting benefits in managing metabolic diseases.

## Highlights

• FASN deletion in POMC neurons causes anorexia and adiposity reduction.
• FASN deficiency increases energy expenditure in a development-dependent manner.
• FASN deficiency increases baseline activity of POMC neurons.
• Adult FASN deletion in POMC neurons increases carbohydrate utilization.
• Adult FASN deficiency or POMC activation limits insulin release via adrenergic signals.

## Graphical abstract

**Figure:**
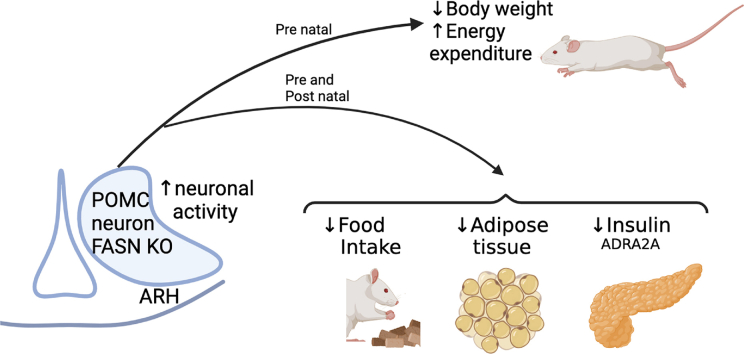

## 1 Introduction

Inhibitors of Fatty Acid Synthase (FASN) such as cerulenin or C75 are known to cause anorexia and significant body weight loss [1]. FASN, a key enzyme catalyzing the first critical step in fatty acids (FA) synthesis, is ubiquitously expressed, including the central nervous system [2,3]. Pharmacological and genetic studies indicate that the anorectic effects of FASN blockade are centrally mediated [4,5], suggesting that FASN may function as a metabolic sensor in various hypothalamic neurons, including populations located in the arcuate nucleus (ARH) or the ventromedial nucleus of the hypothalamus (VMH) [6,7]. However, to our knowledge*,* the effects of specific FASN deletion in any of these populations remain untested.

In the hypothalamus, the Pro-Opiomelanocortin (POMC) neurons in the ARH are critical regulators of energy balance [[8], [9], [10]]. These neurons are activated in states of positive energy balance, decreasing food intake and increasing energy expenditure primarily through the release of alpha-Melanocyte-Stimulating Hormone (a-MSH), which binds to the melanocortin receptor 4 (MC4R) in the post synaptic neurons [[10], [11], [12]]. Dysfunction of POMC neurons or the MC4R leads to severe obesity and associated metabolic comorbidities [10,12,13]. Beyond appetite regulation, POMC neurons also modulate insulin and glucose levels to maintain energy homeostasis [14,15].

Interestingly, FASN blockers predominantly activate the lateral ARH, where POMC neurons are located [16,17], mimicking the activation patterns observed after refeeding or leptin administration to signal satiety [18,19]. This raises the possibility that FASN serves as a signal in POMC neurons to control feeding and energy expenditure.

To test this hypothesis, we generated mouse models with FASN selectively deleted in POMC neurons either prenatally or in adulthood. Our results indicate that FASN deficiency increases basal activity of the POMC neurons, leading to reduced food intake and adiposity in both models. These modifications are accompanied by a metabolic shift in nutrient utilization, driven by modifications in insulin secretion and sympathetic output.

Overall, our results emphasize the critical role of FASN in POMC neurons in regulating feeding behavior, energy expenditure and insulin dynamics.

## 2 Results

### 2.1 Constitutive FASN deficiency in POMC neurons reduces food intake and increases energy expenditure

**Figure 1:**
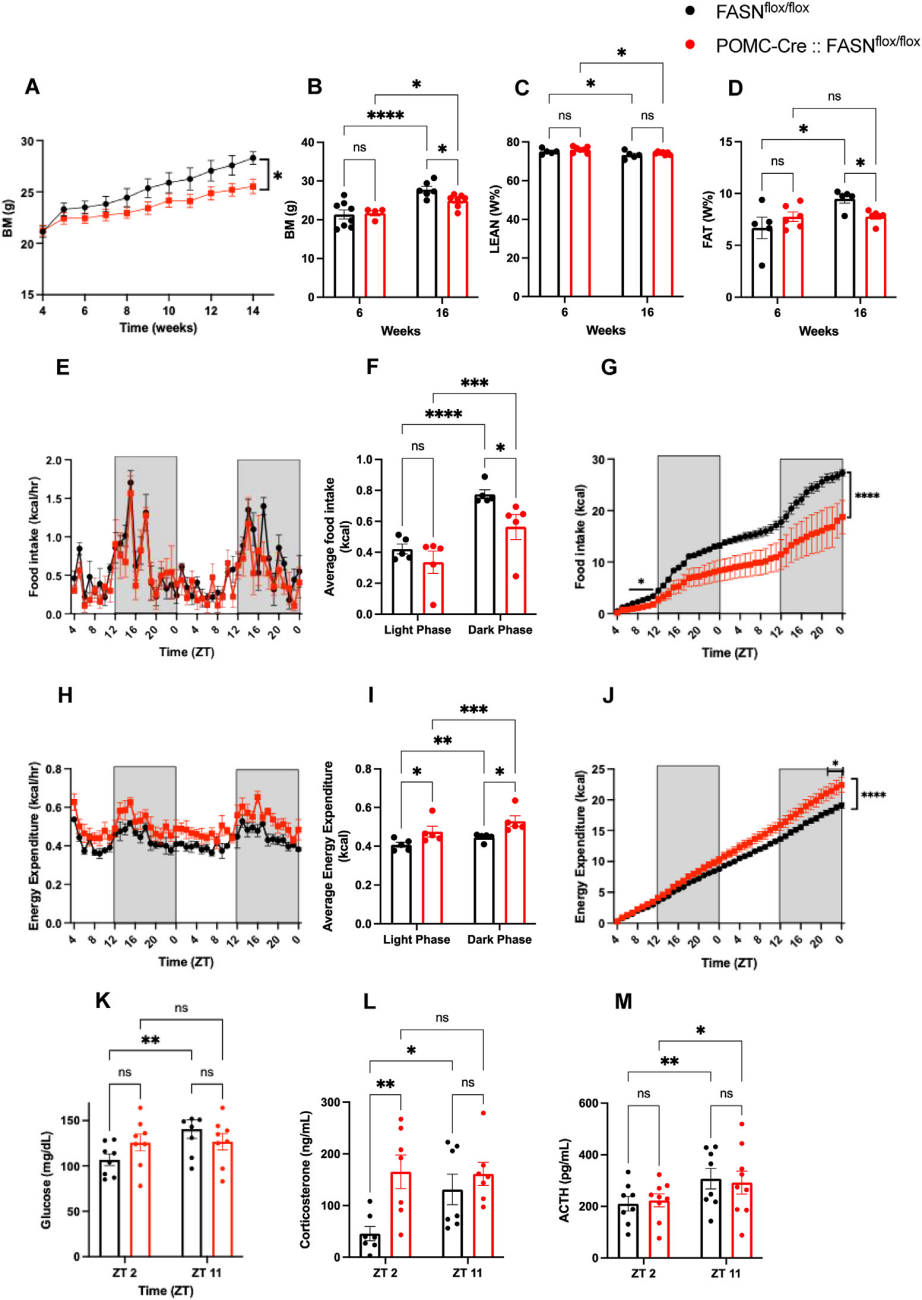
**FASN deletion in POMC neurons produces a lean phenotype.** (A–D) Analysis of body composition. (A) Body weight curve from male mice from 5 to 15 weeks old (Time x Genotype F (9, 99) = 4.883, P < 0.0001, Time F (1.078, 11.86) = 103.7, P < 0.0001) (n = 6, 7). (B–D) Body composition after weaning and 16 weeks old. (B) Total body mass (Time x Genotype F (1, 18) = 16.32, P = 0.0008, Time F (1, 18) = 35.94 P < 0.0001, (C) percentages of lean mass (Time x Genotype F (1, 9) = 0.08991 P = 0.7711, Time F (1, 9) = 14.28 P = 0.0044) and (D) fat mass (Time x Genotype F (1, 9) = 5.448 P = 0.0445, Time F (1, 9) = 5.500 P = 0.0436) (n = 5, 6). (E–G) Food intake measured by metabolic chambers.(E) Food intake pattern (Time x Genotype F (44, 352) = 0.8503 P = 0.7396, Time F (44.00, 352.0) = 8.242 P < 0.0001). (F) Average food intake in the light phase (ZT 0 - ZT 12) and dark phase (ZT 12 – ZT 24) (Time x Genotype F (1, 8) = 3.944 P = 0.0823, Time F (1, 8) = 85.28 P < 0.0001) (G) Cumulative food intake (Time x Genotype F (44, 352) = 4.358 P < 0.0001, Time F (44.00, 352.0) = 104.0 P < 0.0001) (n = 5, 5). (H–J) Analysis of the energy expenditure. (H) Energy expenditure pattern (Time x Genotype F (44, 352) = 0.9832 P = 0.5064, Time F (44.00, 352.0) = 8.254 P < 0.0001). (I) Average energy expenditure in the light and dark phase (Time x Genotype F (1, 8) = 2.098 P = 0.1856, Time F (1, 8) = 68.37 P < 0.0001). (J) Cumulative energy expenditure (Time x Genotype F (44, 352) = 6.373 P < 0.0001, Time F (44.00, 352.0) = 981.4 P < 0.0001) (n = 5, 5). (K–M) Plasma concentrations of (K) glucose (ZT x Genotype F (1, 14) = 6.688 P = 0.0215, ZT F (1, 14) = 7.527 P = 0.0158) (n = 8, 8), (L) corticosterone (ZT x Genotype F (1, 12) = 4.681 P = 0.0514 ZT F (1, 12) = 3.853 P = 0.0733) (n = 7, 7) and (M) ACTH measured at ZT2 and ZT 11 (ZT x Genotype F(0.4289) P = 0.4714, ZT 15.45 P = 0.0005) (n = 8, 8). Data are expressed as mean ± SEM. RM two-way ANAOVA. Significance is noted as ∗p < 0.05, ∗∗p < 0.01, ∗∗∗p < 0.001.

**Figure 2:**
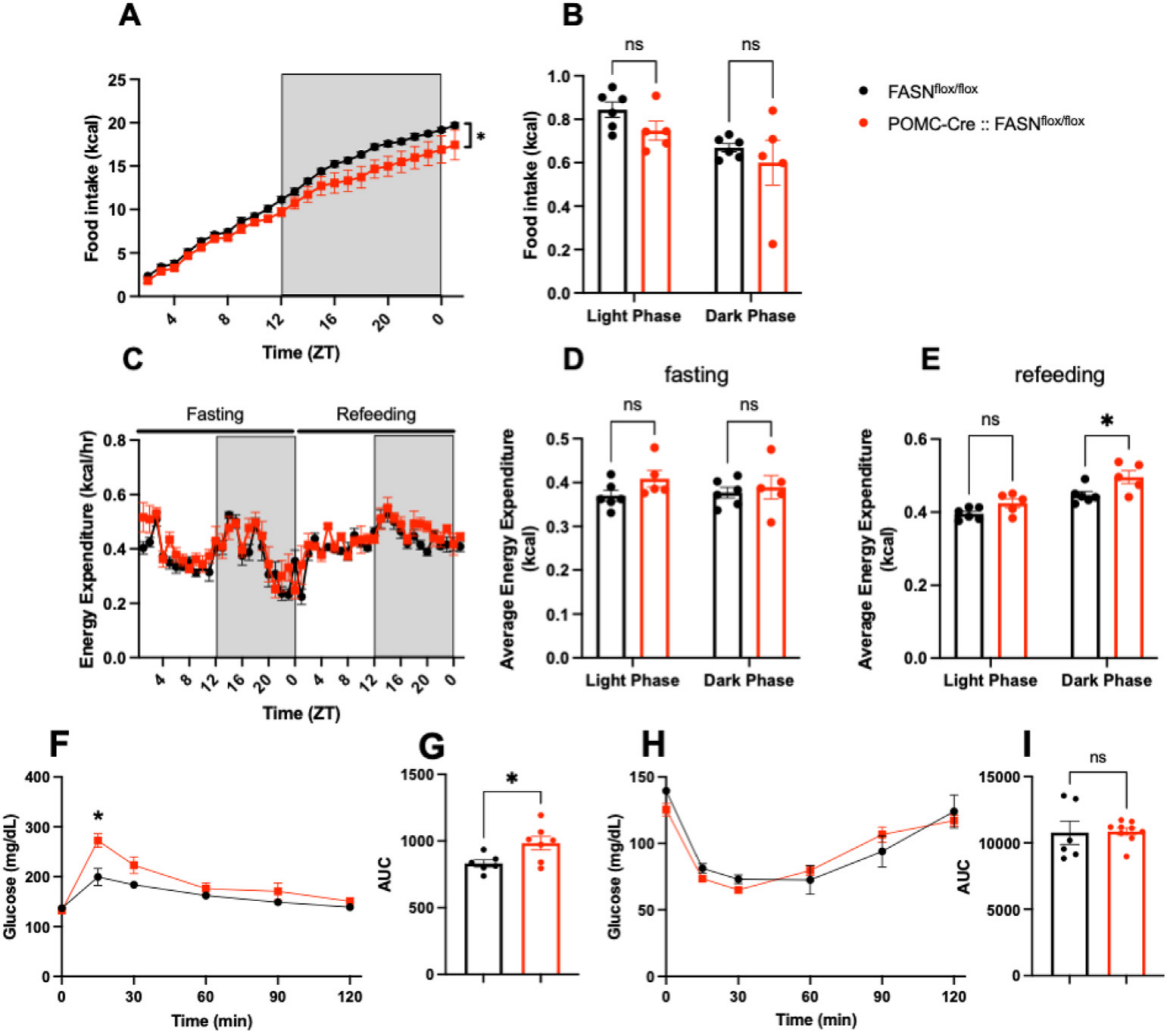
**FASN deletion in POMC neurons decreases food intake after fasting**. Analysis of food intake and energy expenditure by metabolic chambers during 24 h fasting followed by 24 h refeeding.(A, B) Analysis of food intake by metabolic chambers during refeeding. (A) Cumulative food intake during refeeding (Time x Genotype F (23, 207) = 1.774 P = 0.0194, Time F (1.239, 11.15) = 280.6 P < 0.0001). (B) Average food intake in the light and dark phase (Time x Genotype F (1, 9) = 0.08539 P = 0.7767, Time F (1, 9) = 11.74 P = 0.0075) (n = 6, 5). (C–E) Analysis of energy expenditure by metabolic chambers during fasting and refeeding. (C) Comparison of the energy expenditure pattern during 24 h fasting (Time x Genotype F (23, 207) = 1.425 P = 0.1014, Time F (4.237, 38.14) = 12.24 P < 0.0001) and the 24 h after refeeding (Time x Genotype F (24, 216) = 1.256 P = 0.1973, Time F (5.253, 47.28) = 7.929 P < 0.0001). (D) Average energy expenditure in the light and dark phase during fasting (Time x Genotype F (1, 9) = 0.9046 P = 0.3664, Time F (1, 9) = 0.1999 P = 0.6654). (E) Average energy expenditure in the light and dark phase during refeeding (Time x Genotype F (1, 9) = 2.335 P = 0.1608, Time F (1, 9) = 87.31 P < 0.0001) (n = 6, 5). (F–I) Glucose and insulin tolerance tests. (F) GTT Blood glucose (Time x Genotype F (5, 55) = 4.348, P = 0.0021Time F (2.520, 27.72) = 34.78 P < 0.0001) and (G) AUC values after glucose administration (t = 2.512, df = 11 P = 0.0289) (n = 6, 7). (H) ITT blood glucose (Time x Genotype F (5, 65) = 2.164 P = 0.0689, Time F (3.143, 40.86) = 55.70 P < 0.0001) and (I) AUC values after insulin administration (t = 0.1215, df = 13 P = 0.9052) (n = 6, 9). Data are expressed as mean ± SEM. RM two-way ANAOVA, unpaired t test. Significance is noted as ∗p < 0.05, ∗∗p < 0.01, ∗∗∗p < 0.001.

After constitutive deletion of FASN in POMC neurons, 5-week-old POMC-cre::FASN^flox/flox^ male mice displayed no significant differences in body mass or composition compared to the control FASN^flox/flox^ littermates (Figure 1A–D, Figure 2 A-C). By 16-weeks of age, however, POMC-cre::FASN^flox/flox^ mice presented significantly reduced body mass and adiposity relative to controls (Figure 1A–D, Figure 2 A-C). Under ad libitum feeding conditions, POMC-cre::FASN^flox/flox^ mice consumed less food, particularly during the dark phase (Figure 1E–G) and exhibited higher energy expenditure (Figure 1H–J). Notably, locomotor activity (Figure 2 D, E) and RER (Figure 2 F,G) remained similar between groups, suggesting that the lean phenotype results from a combination of increased energy expenditure and decreased food intake.

**Figure 3:**
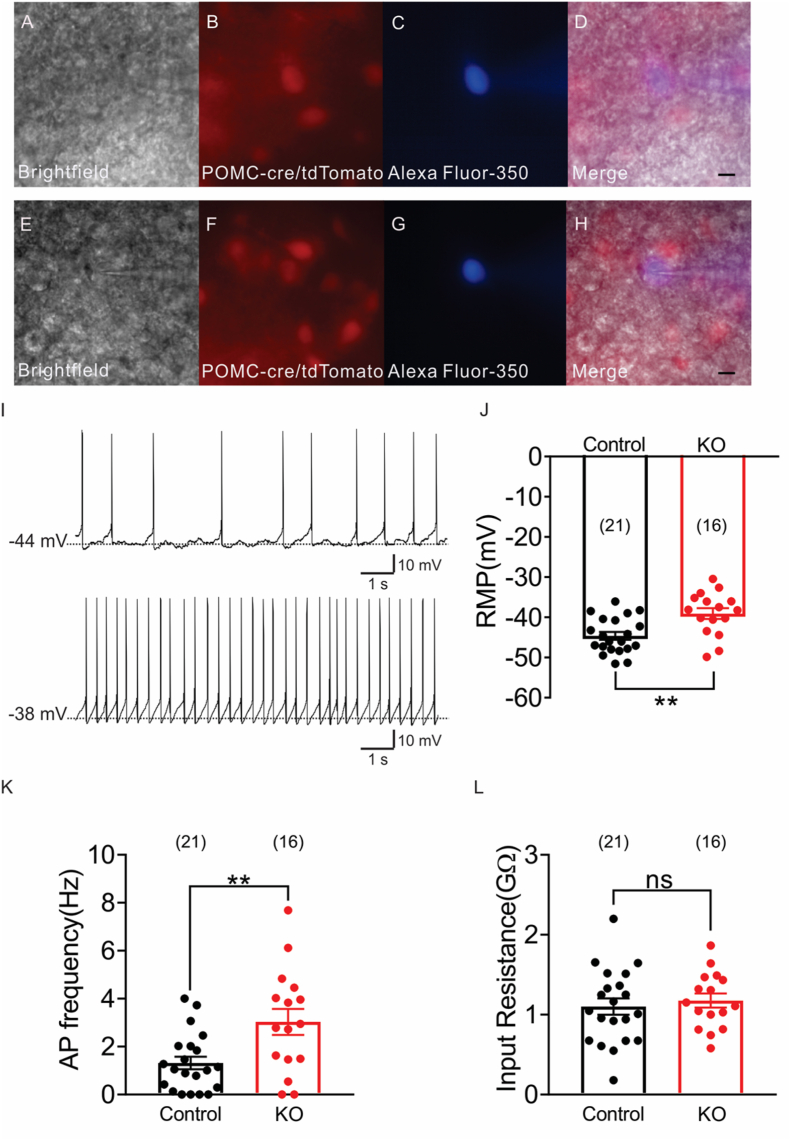
**FASN deficiency increases POMC neuronal activity**. (A–D) Identification of POMC neuron from POMC-Cre::tdtomato mice for recording by (A) Brightfield illumination, (B) TdTomato fluoresce (red), (C) Alexa Fluor 350 hydrazide dye fluorescence (blue). (D)Merged image of targeted POMC neuron. (E–H) Identification of POMC neuron from POMC-Cre::FASN-Flox::tdtomato mice for recording by(E) Brightfield illumination, (F) TdTomato fluorescence, (G) Alexa Fluor 350 hydrazide dye fluorescence. (H)Merged image of targeted POMC neuron.(I) Current-clamp recording of POMC neurons show the resting membrane potential from POMC-Cre::tdtomato control mice and POMC-Cre::FASN-Flox::Tdtomato knockout mice. (J–L) Electrophysiological properties of POMC neurons.(J) Average resting membrane potential (t = 3.424, df = 35 P = 0.0016) (K)Action potential frequency (t = 3.058, df = 35 P = 0.0043) (L)Average input resistance (t = 0.5373, df = 35 P = 0.5944) of POMC neurons from control and knockout mice (n = 21, 16). (A–H) Arrow indicates the targeted cell, scale bar = 50 um. Data are expressed as mean ± SEM. Unpaired t test. Significance is noted as ∗p < 0.05, ∗∗p < 0.01, ∗∗∗p < 0.001. (For interpretation of the references to color in this figure legend, the reader is referred to the Web version of this article.)

While we did not find differences in the circadian patterns of food intake, energy expenditure or locomotor activity (Figure 1 E, H, Figure 2 D, F), POMC-Cre:: FASN^flox/flox^ mice showed higher glucose and corticosterone levels at the beginning of the light phase, with no clear circadian rhythm (Figure 1 K, L). Importantly, ACTH levels were not different, with both groups showing a distinct circadian pattern. (Figure 1 M). Moreover, the pituitary gland of POMC-Cre FASN^flox/flox^::TdTomato mice and control mice showed equally discontinuous fluorescently labeled cells in the anterior lobe, the intermediate lobe was homogeneously labeled, and POMC cells were absent in the posterior lobe [20]. (Figure 3 A,B), suggesting changes on glucose and corticosterone are not due to anatomical changes in the pituitary gland.

**Figure 4:**
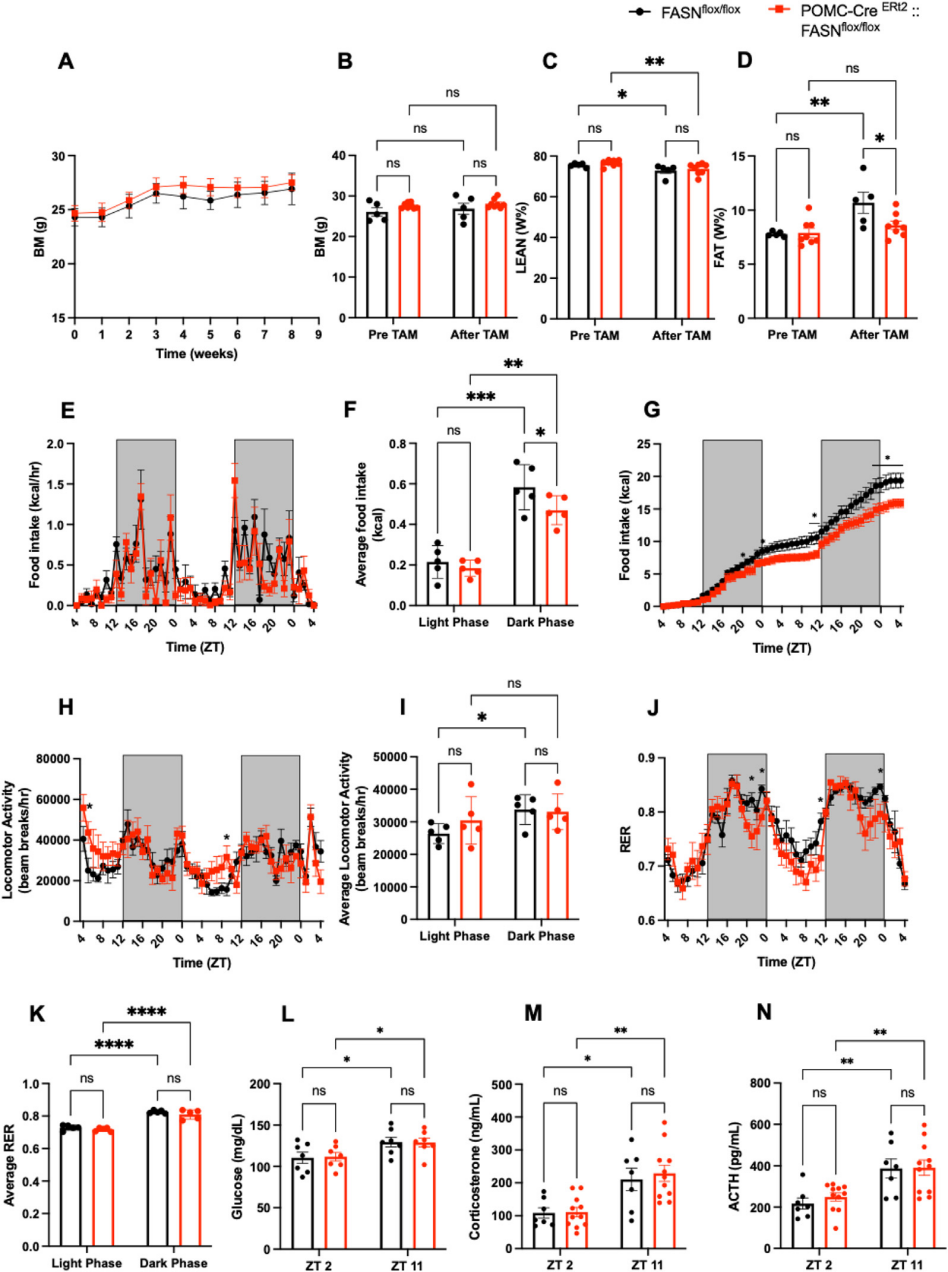
**POMC-Cre ^ERt2^:: FASN^flox/flox^ mice display lower food intake, lower fat mass and changes of circadian patterns.** (A) Body weight curve 8 weeks after recombination (Time x Genotype F (8, 72) = 0.4498 P = 0.8868, Time F (3.003, 27.03) = 23.90 P < 0.0001) (n = 5, 6). (B) Total body mass (Time x Genotype F (1, 11) = 0.1643 P = 0.693, Time F (1, 11) = 2.095 P = 0.1756), (C) percentages of lean mass (Time x Genotype F (1, 11) = 0.01986 P = 0.8905, Time F (1, 11) = 19.44 P = 0.001) and (D) fat mass pre tamoxifen and 8 weeks later by NMR (Time x Genotype F (1, 11) = 6.309 P = 0.0289, Time F (1, 11) = 17.17 P = 0.0016) (n = 5, 8). (E–G) Food intake. (E) food intake pattern (Time x Genotype F (48, 384) = 1.169 P = 0.2146, Time F (6.451, 51.60) = 10.20 P < 0.0001). (F) Average food intake in the light and dark phase (Time x Genotype F (1, 8) = 0.9214 P = 0.3652, Time F (1, 8) = 57.83 P < 0.0001). Genotype F (1, 8) = 7.424 P = 0.0261. (G) Cumulative food intake (Time x Genotype F (48, 384) = 3.423 P < 0.0001, Time F (1.996, 15.97) = 317.8 P < 0.0001) (n = 5, 5). Genotype F (1, 8) = 6.034 P = 0.0395. (H–I) Locomotor activity. (H) Locomotor activity pattern (Time x Genotype F (48, 384) = 1.481 P = 0.0250, Time F (6.904, 55.23) = 5.825 P < 0.0001). (I) Average locomotor activity in the light and dark phase (Time x Genotype F (1, 8) = 2.171 P = 0.1789, Time F (1, 8) = 9.951 P = 0.0135). (J–K) Analysis of the RER. (J) RER pattern (Time x Genotype F (48, 384) = 1.324 P = 0.0812, Time F (5.111, 40.89) = 18.63 P < 0.0001). (K) Average RER in the light and dark phase (Time x Genotype F (1, 8) = 0.1705 P = 0.6905, Time F (1, 8) = 51.06 P < 0.0001) (n = 5, 5). (L–N) Plasma concentrations of (L) glucose (Time x Genotype F (1, 12) = 0.03491 P = 0.8549, Time F (1, 12) = 13.22 P = 0.0034) (n = 7, 7). (M) corticosterone (Time x Genotype F (1, 16) = 0.1118 P = 0.7424, Time F (1, 16) = 21.65 P = 0.0003) (n = 7, 11) and (N) ACTH (Time x Genotype F (1, 32) = 0.1658 P = 0.6866, Time F (1, 32) = 20.89 P < 0.0001) (n = 7, 11). Levels were measured at the begging of the resting phase (ZT2) and before the activity phase (ZT 11). Data are expressed as mean ± SEM. Significance is noted as ∗p < 0.05, ∗∗p < 0.01, ∗∗∗p < 0.001.

We next we challenged the mice with a 24 h fasting and refeeding protocol. During fasting POMC-Cre:: FASN^flox/flox^ mice decreased energy expenditure to levels similar to controls (Figure 2 C, D). However, EE increased again during the dark phase post-refeeding (Figure 2 E), despite lower cumulative food intake (Figure 2 A). We did not observe differences in locomotor activity (Figure 4 A-C) or RER (Figure 4 D-F) during fasting or refeeding.

To investigate the ability of POMC-Cre:: FASN^flox/flox^ mice to control glucose metabolism, we performed glucose and insulin tolerance tests (GTT: 1 g dextrose/kg, IP and ITT: Insulin 0.5 UI/Kg, IP). Interestingly, POMC-Cre FASN^flox/flox^ mice showed higher glucose levels after the GTT (Figure 2 F, G), but no differences in insulin sensitivity compared to controls (Fig. 2H, I). Overall, POMC-cre::FASN^flox/flox^ mice showed higher energy expenditure, a reduction in food intake even after fasting, and altered glucose utilization.

### 2.2 Constitutive deficiency of FASN increases POMC neuron activity

To explore the impact of FASN deletion on POMC neuronal activity, we recorded electrophysiological data from POMC neurons in control (POMC-Cre::TdTomato) and FASN deficient male mice (POMC-Cre:: FASN^flox/flox^::TdTomato). Based on previous evidence linking pharmacological FASN blockade to surrogates of POMC neuronal activation, we hypothesized that FASN knockout would increase POMC neuron activity. Whole-cell patch-clamp recordings were performed from POMC-cre:: TdTomato (21 neurons) and POMC-Cre::FASN ^flox/flox^:: TdTomato mice (16 neurons)) (Figure 3A–H).

Control POMC neurons had on average a resting membrane potential of −44.6 ± 0.9mv, action potential frequency of 1.3 ± 0.3 Hz, input resistance of 1.1 ± 0.1 GΩ and overshooting action potentials (n = 21; Figure 1I–L).

In comparison, FASN deficient POMC neurons exhibited depolarized resting membrane potential (−39.1 ± 1.3mv, Figure 3 J) and increased action potential frequency (3.0 ± 0.5 Hz, Figure 3 K) without changes in input resistance (1.1 ± 0.1 GΩ, Figure 3 L. These data indicate that FASN deficiency significantly increased basal activity of POMC neurons.

### 2.3 Adult deletion of FASN in POMC neurons reduces food intake and adiposity and promotes carbohydrate utilization in response to exogenous glucose

To further delineate the role of FASN unambiguously in POMC neurons in metabolic control, we opted for breeding FASN^flox/flox^ mice to a tamoxifen-inducible POMC-cre mouse model (POMC-Cre^ERt2^) to continue our experiments. This model allowed us to precisely regulate Cre recombinase activity for adult deletion of FASN, avoiding unintended effects in off-target neuronal populations, plasticity in the neuronal circuits or adaptations to chronic metabolic changes in the body inherent to the constitutive models.

**Figure 5:**
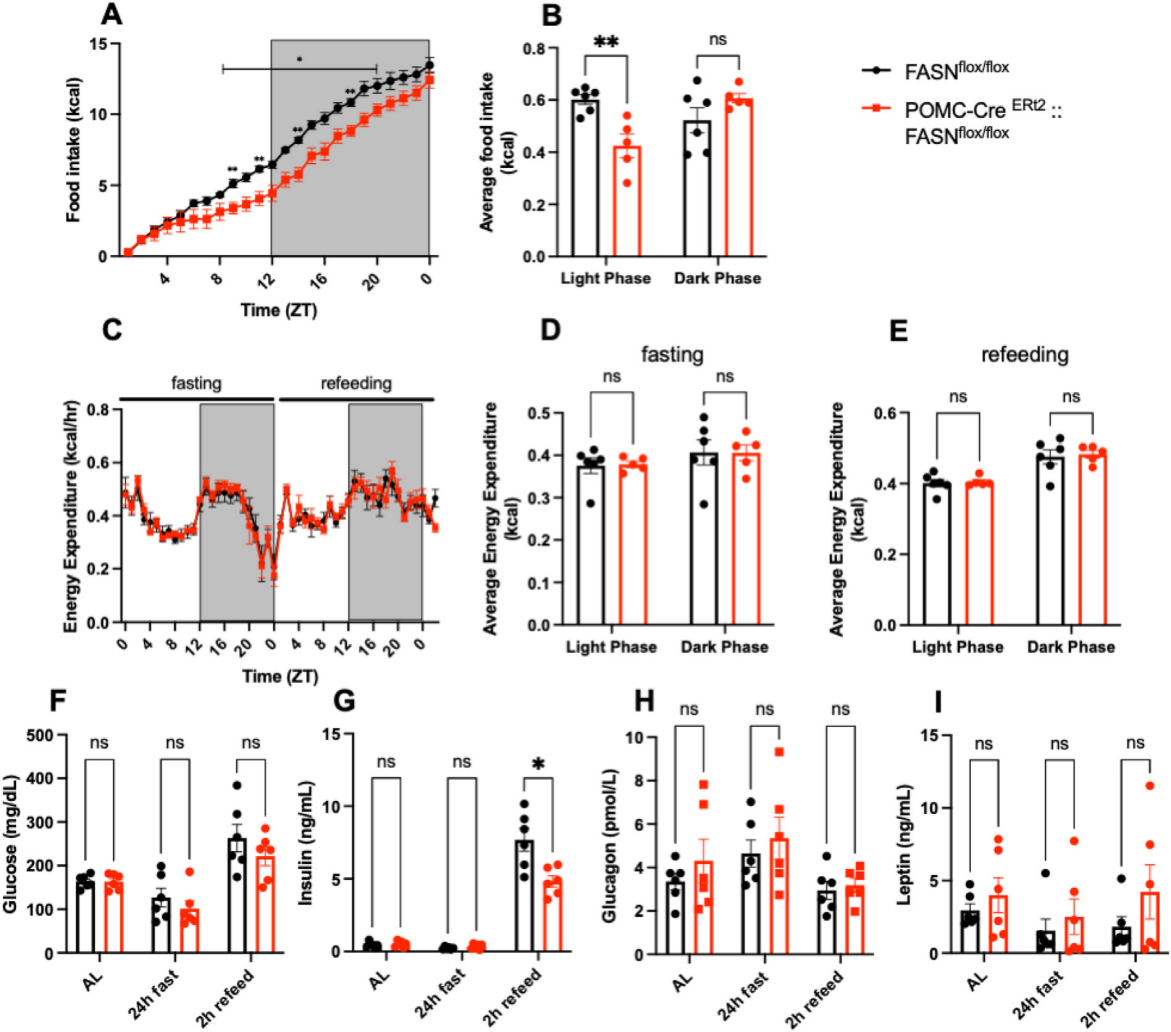
**POMC-Cre^ERt2^:: FASN^flox/flox^ mice have reduced food intake after fasting and modified postprandial metabolic profile**. Analysis of food intake and energy expenditure by metabolic chambers during 24 h fasting followed by 24 h refeeding (A, B). Analysis of food intake by metabolic chambers during refeeding. (A) Cumulative food intake during refeeding (Time x Genotype F (23, 207) = 3.823 P < 0.0001, Time F (3.356, 30.20) = 404.6 P < 0.0001). (B) Average food intake in the light and dark phase (Time x Genotype F (1, 9) = 12.28 P = 0.0067 Time F (1, 9) = 1.882 P = 0.2033) (n = 6, 5). (C–E) Analysis of the energy expenditure. (C) Comparison of the energy expenditure pattern during 24 h fasting (Time x Genotype F (24, 216) = 0.4302 P = 0.9916, Time F (4.531, 40.78) = 24.39 P < 0.0001) and the 24 h after refeeding (Time x Genotype F (23, 207) = 0.5430 P = 0.9575, Time F (6.805, 61.25) = 6.943 P < 0.0001). (D) Average energy expenditure in the light and dark phase during fasting (Time x Genotype F (1, 9) = 0.02658 P = 0.8741, Time F (1, 9) = 6.439 P = 0.0318). (E) Average energy expenditure in the light and dark phase during refeeding (Time x Genotype F (1, 9) = 0.03304 P = 0.8598, Time F (1, 9) = 82.51 P < 0.0001)(n = 6, 5). (F–I) Plasma concentrations of (F) glucose (Time x Genotype F (2, 20) = 0.5901 P = 0.5637, Time F (1.725, 17.25) = 23.30 P < 0.0001), (G) insulin (Time x Genotype F (2, 20) = 10.99 P = 0.0006, Time F (1.017, 10.17) = 173.7 P < 0.0001), (H) glucagon (Time x Genotype F (2, 20) = 1.173 P = 0.3298, Time F (1.436, 14.36) = 11.34 P = 0.0022) and (I) leptin (Time x Genotype F (2, 20) = 1.087 P = 0.3564, Time F (1.589, 15.89) = 3.646 P = 0.0583) (n = 6, 6) ad libitum, after 24 h fasting and 2 h after refeeding. Data are expressed as mean ± SEM. RM two-way ANOVA, Significance is noted as ∗p < 0.05, ∗∗p < 0.01, ∗∗∗p < 0.001**.**

**Figure 6:**
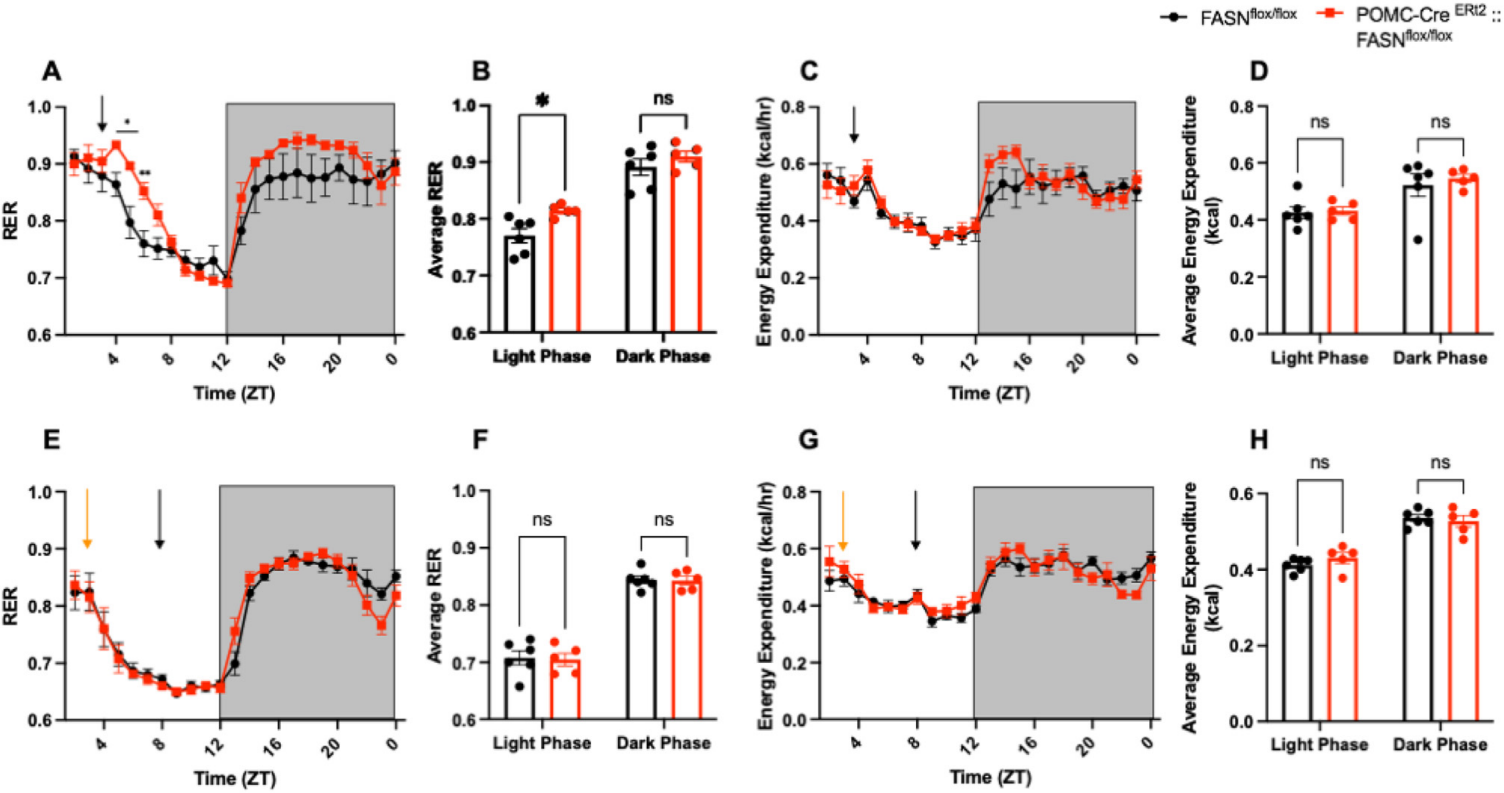
**POMC-Cre ^ERt2^:: FASN ^flox/flox^ mice increase carbohydrate utilization in response to exogenous glucose**. Analysis of energy expenditure and RER after carbohydrate or lipid administration.(A–D) Metabolic response to exogenous glucose. (A) Comparison of the RER pattern (Time x Genotype F (23, 207) = 1.691 P = 0.0295, Time F (2.703, 24.32) = 26.80 P < 0.0001) (B) Average RER in the light phase (ZT 0 - ZT 12) and dark phase (ZT 12 – ZT 24) (Time x Genotype F (1, 9) = 0.8313 P = 0.3857, Time F (1, 9) = 58.31 P < 0.0001).Genotype F (1, 9) = 13.29 P = 0.0054.(C) Comparison of the energy expenditure pattern (Time x Genotype F (23, 207) = 1.165 P = 0.2801, Time F (4.137, 37.24) = 12.86 P < 0.0001)(D) Average energy expenditure in the light phase and dark phase (Time x Genotype F (1, 9) = 0.1247 P = 0.7321, Time F (1, 9) = 16.35 P = 0.0029) (n = 6, 5). (E–H) Metabolic response to lipids. (E) Comparison of the RER pattern (Time x Genotype F (22, 198) = 1.179 P = 0.2703, Time F (3.610, 32.49) = 75.16 P < 0.0001) (F) Average RER in the light phase and dark phase (Time x Genotype F (1, 9) = 0.01939, P = 0.8923, Time F (1, 9) = 348.7 P < 0.0001). (G) Comparison of the energy expenditure pattern (Time x Genotype F (22, 198) = 1.093 P = 0.3569, Time F (5.215, 46.93) = 16.09 P < 0.0001) (H) Average energy expenditure in the light phase and dark phase (Time x Genotype F (1, 9) = 1.481 P = 0.2545, Time F (1, 9) = 96.07 P < 0.0001) (n = 6, 5). Data are expressed as mean ± SEM. RM two-way ANOVA. Significance is noted as ∗p < 0.05, ∗∗p < 0.01, ∗∗∗p < 0.001. Black arrows indicate glucose administration (A, C), or Intralipid administration (E, G), Orange arrows indicate beginning of fasting (E, G). (For interpretation of the references to color in this figure legend, the reader is referred to the Web version of this article.)

After Tamoxifen administration, we used RNAscope fluorescent in situ hybridization to verify the colocalization of POMC and FASN mRNAs. As expected, FASN mRNA was present in the majority of POMC neurons in control mice (Figure 5 A-D, L), while POMC and FASN mRNAs minimally colocalized in the ARH of POMC-Cre^Ert2^:: FASN^flox/flox^ mice (Figure 5 E-H, M, N), confirming the successful recombination and deletion of FASN from the majority of POMC neurons. No anatomical differences were observed at the level of the pituitary gland between POMC-Cre^ERt2^:: FASN^flox/flox^:: ZsGreen mice compared to POMC-Cre^ERt2^:: ZsGreen mice. Both groups presented homogeneous fluorescence in the intermediate lobe, sparce cells in the anterior lobe, and no fluorescence in the posterior lobe (Figure 3 A, C). Furthermore, the qPCR analysis of gene expression in the ARH confirmed a statistically significant reduction of FASN mRNA in the POMC-Cre^ER t2^:: FASN^flox/flox^ mice compared to their control FASN^flox/flox^ littermates (Figure 6 A). In addition, we found lower expression of hypothalamic PPARa (Figure 6 B). POMC (Figure 6 C) CART (Figure 6 D) AgRP (Figure 6 E) and NPY(Figure 6 F) gene expression was not different between groups.

**Figure 7:**
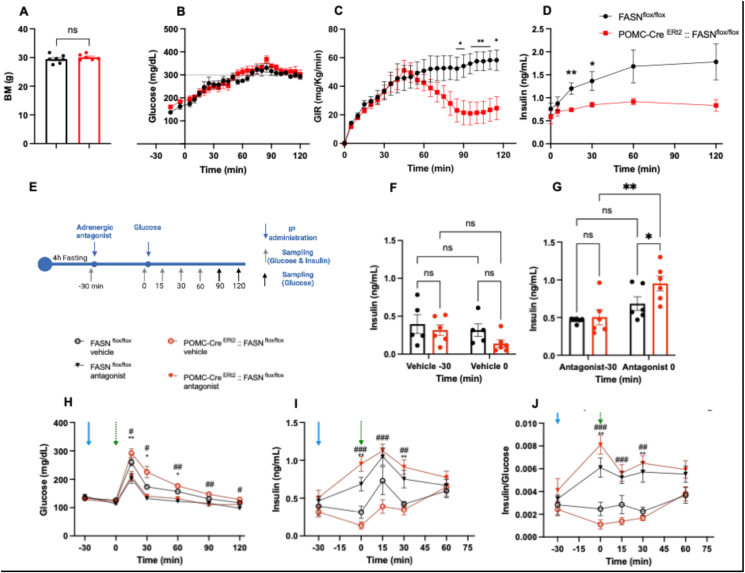
**FASN in POMC neurons is required for glucose-induced insulin release and is mediated by adrenergic signals.** (A–D) Analysis of insulin release in response to continuous glucose infusion. (A) Body mass before glucose infusion (t = 0.9094, df = 11 P = 0.3826). (B) Blood glucose levels (Time x Genotype F (25, 275) = 0.6151 P = 0.9268, Time F (2.337, 25.71) = 32.43 P < 0.0001). (C) Glucose infusion rate (Time x Genotype F (23, 253) = 4.377 P < 0.0001, Time F (2.494, 27.43) = 11.18 P = 0.0001). (D) Insulin concentrations (Time x Genotype F (5, 55) = 2.418 P = 0.0472, Time F (2.315, 25.47) = 6.837 P = 0.0030) (n = 7, 6) Genotype F (1, 11) = 5.375 P = 0.0407. (E–F) Response to the A2 adrenergic receptor antagonist atipamezole (E) Schematic representation of the experimental design. (F) Insulin concentrations after vehicle (Time x Genotype F (1, 9) = 0.5137 P = 0.4917, Time F (1, 9) = 3.625 P = 0.0893 (n = 5, 6) or (G) adrenergic antagonist administration (Time x Genotype F (1, 10) = 1.872 P = 0.2012, Time F (1, 10) = 16.22, P = 0.0024) (n = 6, 6). (H) Blood Glucose (Time x Genotype F (18, 114) = 4.996, P < 0.0001, Time F (2.348, 44.61) = 123.1, P < 0.0001 Genotype F (3, 19) = 14.59 P < 0.0001), (I) Insulin (Time x Genotype F (12, 76) = 4.254 P < 0.0001Time F (3.090, 58.71) = 17.08 P < 0.0001Genotype F (3, 19) = 10.68 P = 0.0002) and (J) Insulin-to-glucose ratio (Time x Genotype F (12, 76) = 4.321 P < 0.0001, Time F (3.073, 58.38) = 5.662 P = 0.0017 Genotype F (3, 19) = 14.36 P < 0.0001) (n = 5, 6, 6, 6) in response to glucose administration after pretreatment with the adrenergic antagonist. Data are expressed as mean ± SEM. Unpaired t test, RM two-way ANOVA, Significance is noted as ∗p < 0.05, ∗∗p < 0.01, ∗∗∗p < 0.001 from FASN^flox/flox^ (vehicle) or #p < 0.05, ##p < 0.01, ###p < 0.001 from POMC-Cre ^Ert2^:: FASN ^flox/flox^ (vehicle)**.** The arrows indicate IP administration of adrenergic antagonist (blue), or glucose (green). (For interpretation of the references to color in this figure legend, the reader is referred to the Web version of this article.)

Before recombination, both groups had equivalent body weight, lean mass, and fat mass (Figure 4. B-D). 8 weeks after tamoxifen administration, male FASN deficient mice showed noticeable reduction of fat accumulation (Figure 4C, D), confirmed by smaller perigonadal and perirenal adipose tissue (Figure 7 B, C). However, we did not find major differences in total body mass (Figure 4 A, B) or BAT, subcutaneous adipose tissue or liver mass in proportion to body mass (Figure 7 A, D, E).

To further study the mechanisms underlying the lean phenotype, we placed the mice in the metabolic chambers 5 days post tamoxifen administration, before any significant changes of body composition were observed. When fed ad libitum, POMC-Cre^ERt2^:: FASN^flox/flox^ mice experienced a reduction of total food intake, particularly during the dark phase (Figure 4 F, G). While no differences were observed in energy expenditure (Fig. S7 H-I), average locomotor activity or RER between groups, the hourly patterns of locomotor activity and RER were altered in the POMC-Cre^ERt2^:: FASN^flox/flox^ mice (Figure 4I–K).

Corticosterone and ACTH in the bloodstream had higher levels at ZT 11, before the activity onset, compared to ZT 2 (Figure 4L–N). Therefore, adult deletion of FASN in POMC cells did not change the daily pattern, implying that the changes corticosterone observed in the constitutive model at the beginning of the light phase were likely due to developmental expression of POMC. We did not find differences in the daily glucose pattern, or the expression of genes involved in gluconeogenesis in the liver, G6PC (Figure 6 G), PEPCK (Fig. S6 H), FBP1(Figure 6 I), PC (Figure 6 J)or GSK (Figure 6 L) in response to FASN deletion in the adulthood. Expression of GCK was downregulated as expected when insulin levels are low [21] (Figure 6 K).

**Figure 8:**
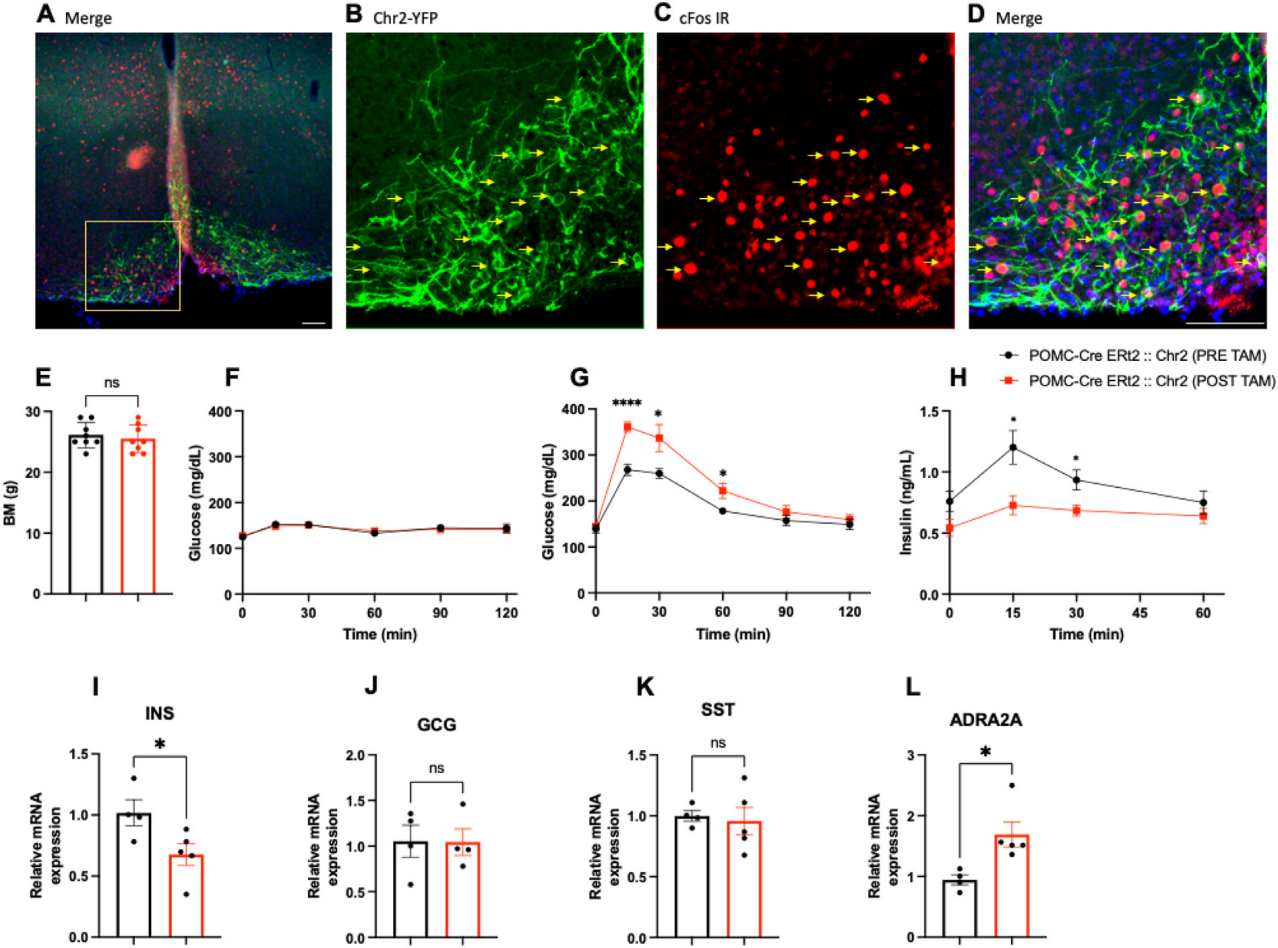
**Optogenetic stimulation of POMC neurons diminishes insulin secretion.** (A–D) Fluorescent immunohistochemistry shows POMC neurons expressing eGFP (green) and cFos (red) after optogenetic stimulation. Yellow arrows indicate activated POMC neurons. (E) Body mass before and ine week post recombination (STATS). (F) Glucose levels during optogenetic stimulation of POMC neurons (Time x Genotype F (5, 70) = 1.028 P = 0.4082 Time, F (2.514, 35.20) = 3.276 P = 0.0395) (n = 8, 8). (G) Glucose (Time x Genotype F (5, 70) = 5.457 P = 0.0003, Time F (2.709, 37.93) = 92.02 P < 0.0001) Genotype F (1, 14) = 10.03 P = 0.0069 (n = 8, 8) and (H) Insulin levels (Time x Genotype F (3, 42) = 2.891 P = 0.0465, Time F (3, 42) = 9.731 P < 0.0001) Genotype F (1, 14) = 7.993 P = 0.0134) during optogenetic stimulation concomitant with a GTT performed before and one week after recombination. (I–L) Relative mRNA expression in the pancreas after optogenetic stimulation (I) INS (t = 2.467, df = 7 P = 0.043), (J) CGC (t = 0.04145, df = 6 P = 0.9683), (K) SST (t = 0.3154, df = 7 P = 0.7617) and (L) ADRA2A (t = 3.073, df = 7 P = 0.018) (n = 8, 8). Scale bar 100 um. Data are expressed as mean ± SEM. RM two-way ANOVA, Unpaired t test, ∗P < 0.05, ∗∗p < 0.01. experiment is as follows: mice received a dose of a drug or vehicle, 30 min later performed a GTT to evaluate the glucose and insulin response results show higher glucose values and lower insulin release in response to glucose administration after the drug, measurements were taken right before the drug was given, then glucose was given 30 min later, and measurements were taken at 0, 15, 30, 60, 90, and 120 min after glucose administration. all timepoints have glucose and insulin measurements, best option to report the changes. (For interpretation of the references to color in this figure legend, the reader is referred to the Web version of this article.)

When we challenged the mice by 24 h fasting and refeeding, POMC-Cre^ERt2^:: FASN^flox/flox^ mice consumed smaller amounts of food, even after energy deficiency (Figure 5 A, B). However, both groups had a similar energy expenditure (Figure 5. C-E), locomotor activity (Fig S8 A-C) and RER (Fig S8 D-F) during fasting and refeeding. In an independent cohort, we evaluated the changes in glucose homeostasis in response to fasting and refeeding. Strikingly, although POMC-Cre^ERt2^ FASN^flox/flox^ mice consumed significantly less food than FASN^flox/flox^ mice, both groups maintained similar glycemia during the fasting and refeeding phases (Fig. 5F). Moreover, the secretion of insulin was significantly lower after feeding compared to their control littermates (Figure 5 G), while glucagon (Figure 5H) and leptin values were comparable to the control mice (Figure 5 I). When GTT was performed, POMC-Cre^ERt2^ FASN^flox/flox^ showed increased blood glucose (Figure 8 G, H), however, the response to exogenous insulin was in the same range as the control group (Figure 8 I, J).

Based on the on the previous results, we decided to further investigate the metabolic response to exogenous carbohydrates as opposed to exogenous lipids. We administered glucose (1 g dextrose/kg) or Intralipid (: 4uL/g intralipid, oral gavage) to male mice and evaluated their metabolic changes using the metabolic chambers. Remarkably, glucose rapidly increased RER for several hours after administration (Figure 6 A, B) without changing the energy expenditure of the experimental mice (Figure 6C,D). In contrast, administration of intralipid did not impact the RER (Figure 6 E, F) or energy expenditure (Figure 6. G-H), suggesting that the effects of FASN deletion are specific for glucose metabolism.

### 2.4 FASN in POMC neurons is required for adequate glucose-induced insulin release

To investigate the impact of adult FASN deletion in POMC neurons on glucose stimulated insulin secretion, we performed a continuous glucose infusion in age and body matched mice (Figure 7 A). We aimed for a sustained hyperglycemic state (300 mg/dL). Constant circulating glucose values were achieved approximately 80 min after beginning the infusion and was maintained for additional 40 min (Figure 7 B).

POMC-Cre^ERt2^ FASN^flox/flox^ mice required significantly lower glucose infusion rate to reach the hyperglycemic state, compared with the control mice. (Figure 7C). It is noteworthy that the POMC-Cre ^ERt2^:: FASN^flox/flox^ mice also secreted significantly less insulin in response to hyperglycemia as early as 15 min after infusion started. The lower in insulin secretion was maintained until the end of the experiment (Figure 7 D).

### 2.5 Insulin hyposecretion is reversed by the adrenergic A2 receptors blockade

Adrenalin controls insulin secretion via the alpha 2 adrenergic receptors. Pharmacological agonism of the alpha 2 adrenergic receptors inhibits glucose induced insulin release form isolated perfused pancreas [22]. Therefore, we hypothesized that the adrenergic signaling underlies the blunted insulin response observed after glucose administration, and blockade of the alpha 2 adrenergic receptor can restore glucose induced insulin release. To test our hypothesis, we administered alpha 2 adrenergic receptor antagonists atipamezole (2 mg/kg, IP) or vehicle followed by glucose administration (IP, 1 g dextrose/kg) to age and BW matched FASN^flox/flox^ or POMC-Cre ^ERt2^:: FASN^flox/flox^.

Prior to the antagonist administration, basal glucose or insulin levels did not differ between groups (Figure 7F–H). Antagonist pretreatment lowered blood glucose during the GTT in both genotypes compared to the vehicle. Notably, the glucose curves following antagonist treatment were equivalent between FASN^flox/flox^ and POMC-Cre ^ERt2^:: FASN^flox/flox^ mice, in contrast to the higher glucose levels observed in POMC-Cre ^ERt2^:: FASN^flox/flox^ mice treated with vehicle compared to their corresponding control mice (Figure 7H, Fig. S8). The POMC-Cre ^ERt2^:: FASN^flox/flox^ mice treated with the vehicle secreted less insulin in response to the same amount of glucose compared to controls, whereas atipamezole pretreatment increased insulin secretion in the POMC-Cre ^ERt2^:: FASN^flox/flox^ mice compared to the FASN^flox/flox^ and mice treated with vehicle (Figure 7 F, G, I). This indicates that the blunted response of insulin in POMC-Cre ^ERt2^:: FASN^flox/flox^ mice is not due to pancreatic insufficiency, but rather to increased sympathetic tone. Analysis of insulin levels relative to systemic glucose revealed a nearly linear relationship in the vehicle treated FASN^flox/flox^ mice and lower insulin to glucose ratio in the vehicle treated POMC-Cre ^ERt2^:: FASN^flox/flox^mice. Importantly, antagonist treatment increased insulin secretion relative to glucose in both genotypes (Fig. 7J).

Finally, while atipamezole caused higher basal GTT insulin levels in the KO mice, post GTT insulin levels converged with the treated KO mice, suggesting no major differences in insulin sensitivity, as previously indicated by the ITT (Figure 7J, fig, S8).

### 2.6 Optogenetic activation of POMC neurons inhibits glucose-induced insulin release

Finally, based on the increased neuronal activity observed after constitutive FASN deficiency, we decided to test if activation of POMC neurons can recapitulate the inhibition of insulin release in response to acute glucose administration. For this purpose, we generated POMC-Cre ^ERt2^:: Chr2 mice and performed a GTT during optogenetic stimulation before and one week after recombination to induce ChR2 expression. Successful optogenetic stimulation of POMC neurons in the ARH was verified by induction of c-Fos protein expression in POMC neurons (Figure 8A–D). We did not find differences in body mass before and after recombination (Fig 8E).

POMC neuronal activation alone did not produce changes of circulating glucose (Figure 8 F). Blood glucose levels were higher during optogenetic activation of POMC neurons after glucose administration (1 g dextrose/kg, IP) (Figure 8 G), however the release of insulin associated to the administration of glucose was reduced (Figure 8H), comparable the POMC-Cre ^ERt2^:: FASN^flox/flox^ mice. Gene expression levels were assessed by qPCR performed on pancreas collected after the experiment. Our data indicates that Ins mRNA was downregulated (Figure 8 I), we did not find changes in GCG or SST mRNA expression (Figure 8 J, K). However, there was an increase of ADRA2A mRNA after POMC optogenetic stimulation (Figure 8 L).

## 3 Discussion

Constitutive FASN deletion in POMC neurons reduced food intake, increased energy expenditure, and lowered body fat by 16 weeks of age in male mice. This was linked to increased activity in FASN-deficient POMC neurons. Adult deletion also reduced food intake and fat accumulation decreasing glucose-induced insulin secretion, a condition reversed by blocking a2 adrenergic receptors. These results highlight the role of FASN in POMC neurons in regulating energy balance and glucose control.

While previous evidence has established a relationship between fatty acids in the hypothalamus and the regulation of the energy state of the animals [23,24], these conclusions come from administration of exogenous FA directly in the CNS [25,26], or by pharmacological blockade of the fatty acid synthesis. Although the administration of FA has been essential to understand the role of FA in the CNS, it also masks the physiological role of FA synthesis pathway locally. Moreover, long chain fatty acids obtained from the diet might have regulated access to the brain due to the blood–brain barrier [[27], [28], [29]]. On the other hand, pharmacological inhibition of FASN lacks specificity to target unique hypothalamic neuronal populations. We tackled these limitations by using constitutive, and tamoxifen induced POMC-Cre mice models to study the role of FASN specifically in POMC neurons and demonstrated the essential role of FASN in POMC for regulating food intake, body composition, and glucose homeostasis.

In agreement with previous literature [1,4,5,17], our constitutive mouse model decreased food intake and developed a lean phenotype in early adulthood. This is analogous to the effects of long term stimulation of POMC neurons by opto or chemogenetics [30,31], possibly mimicking the late feeding phase, when most POMC neurons are reported to be highly active in response to satiety signals [32,33]. In the same sense, we observed increased energy expenditure, mostly during the active phase, comparably to the effects observed after activation of POMC neurons or administration of melanocortin agonists [34]. This increase is dependent on the metabolic state of the animal, as fasting, when POMC neurons are mostly silenced and therefore a-MSH release is suppressed, eliminated the differences on energy expenditure.

In our hands, POMC neurons have elevated resting membrane potential and increased firing rate when lacking FASN. This finding is consistent with studies using pharmacological inhibitors and histological approaches showing increased neuronal activation markers in putative POMC neurons [16,17,35]. Furthermore, electrophysiological recordings indicate that long chain fatty acids have an inhibitory effect on POMC neuronal activity [36]. Therefore, deficiency of fatty acids could lead to more active POMC neurons. To our knowledge, this is the first time to show that deficiency of FA neuronal synthesis changes neuronal electrical properties, indicating that the phenotype observed is associated to elevated POMC neuronal activity.

It is important to note, however, that metabolic effects were accompanied by a dysregulation of the corticosterone release in the prenatal model. Mice lacking FASN during development exhibited constant higher Cort, however, we did not observe differences in ACTH values. Therefore, it is possible that changes of Cort release and some other metabolic effects are driven in part by the pre autonomic output from other neuronal sub populations derived from POMC progenitors, as it is now recognized that pre or postnatal recombination in POMC neurons causes different phenotypes [37,38]. For example, single cell sequencing experiments done with POMC-eGFP mice demonstrated that approximately 28% POMC cells later develop into AgRP/NPY neurons [39]. AgRP neurons, in turn, can regulate corticosterone secretion [[40], [41], [42]]. Further investigation is needed to clarify if this phenomenon is due to neuronal plasticity or developmental off-target of neurons derived from the POMC lineage. For these reasons, we decided to evaluate the effects of FASN genetic deficiency in adulthood by taking advantage of a tamoxifen-inducible PomcCre^ERt2^ transgenic mouse model.

Consistent with the developmental model, FASN deletion in adulthood reduced food intake and adiposity. Intriguingly, we did not find differences in energy expenditure or endogenous levels of glucose or corticosterone compared to the control littermates. We hypothesized that different outcomes may be attributed to more controlled recombination in *bona fide* POMC neurons. Additionally, previous evidence suggests that the melanocortin system uses independent pathways to regulate either food intake or energy expenditure. Specifically, food intake is regulated via the PVH, while energy expenditure is distributed into other nuclei [43]. During development, FASN deficiency could affect multiple neuronal outputs targeting areas beyond the PVH, consequently increasing basal energy expenditure [44]. In contrast, the effects of adult deletion may be more restricted to a sub population of POMC neurons projecting to the PVH [19,45], minimizing changes in energy expenditure, and mainly affecting food intake and glucose metabolism. The identification of POMC neuronal subpopulations responsive almost exclusively to leptin, insulin or serotonin supports the notion of specific metabolic functions allocated in different POMC neurons [46,47]. Similarly, recent genetic intersectional approaches found that activation of POMC-GLP1r neurons decreases food intake, while acute stimulation of POMC-LepR neurons has minimal effects on feeding [48].

While the pituitary, one of the organs with the highest POMC expression, did not show evident changes in morphology or tissue organization, it remains uncertain whether the hypothalamus is similarly unaffected. Potential neuronal alterations associated to FASN deficiency could occur at different levels: survival [49], fate specification [50], or neuronal projections [51]. Addressing these questions would require advanced techniques such as single-cell sequencing and light-sheet microscopy. However, previous studies suggest that global hypothalamic FASN deletion does not impair neuronal survival [5], as it would be incompatible with life, and POMC defiency leads to obesity-related phenotypes, arguing against major developmental disruptions in POMC neurons. Although less likely, the increased neuronal activity observed could in part reflect a compensatory mechanism to decreased neuronal number or output, analogous to other neuroadaptive responses seen in conditions like Parkinson's disease. Intracellularly, inhibition of FASN by cerulenin or C75 decreases palmitate levels, while Acetyl-CoA and malonyl-CoA levels are elevated [52,53]. The accumulation of malonyl-CoA in turn inhibits the translocation of FAs in the mitochondria by allosteric inhibition of the Carnitine Palmitoyl transferase 1(CPT1) [54]. However, the regulation and effects of FASN in specific POMC neuronal subpopulations is not characterized and requires further research.

The increased activity of POMC neurons may reduce insulin release via MC4R signaling. Deletion of MC4R leads to hyperinsulinemia prior to the onset of obesity [15,55] while central administration of melanocortin agonists increases the sympathetic tone [56] and decreases serum insulin levels [15] demonstrating that the melanocortin activity is necessary and sufficient for the adequate balance of insulin secretion. Therefore, exposure to a substantial bolus of glucose may result in an impaired glucose tolerance test due to the amplified activity of the melanocortin system to limit insulin release.

In *ex vivo* models, alpha 2 adrenergic agonists inhibit glucose-induced insulin release form the isolated perfused rat pancreas [22]. Similarly, polymorphisms or over-expression of the gene ADRA2A encoding the adrenergic receptor alpha 2 A in humans have been associated with reduced insulin secretion [57], and is corrected by administration of alpha 2 A AR antagonists [58]. In our hands, blockade of the alpha 2 adrenergic receptor normalized insulin release after glucose administration, implying that FASN deletion in POMC neurons chronically augmented the sympathetic tone, later translated to reduced adiposity. The fact that circulating leptin was similar between groups despite the difference in adiposity or food intake agrees with previous demonstrations of leptin regulation by adrenergic signals in parallel to changes in adiposity [38] and suggests that the sympathetic changes might extend to adipose tissue. Previous works have shown that stimulation of POMC neurons influences hepatic metabolism [59]. We did not find evidence of constitutive changes in gluconeogenesis-related genes. However, we did find changes in GCK in a manner consistent with lower insulinemia [21]. Insulin regulates GCK acutely by inhibiting FOXO1-mediated repression and sustaining its expression through SREBP-1c activation. Since mice consumed food until the end of the dark phase, samples collected between ZT 0 and ZT 2 may still reflect postprandial effects, providing a physiological context where the liver responds to lower insulin levels after extended nocturnal feeding.

In our study, experimental mice achieved postprandial blood glucose levels comparable to the control group with reduced insulin release, indicating improved insulin sensitivity. Similarly, the results from the metabolic chambers demonstrate that a single challenge of glucose administration leads to a sustained elevation of the RER, suggesting improved use of glucose as an energy source [60,61]. However, it is important to consider that signs of improved insulin sensitivity come from experiments triggering endogenous insulin secretion, as the response to exogenous insulin remained similar between groups.

Targeting FA synthesis in POMC neurons may help prevent hyperinsulinemia and fat accumulation. Moreover, FASN inhibitors like TVB-2640 [62], IPI-9119 [63], GSK2194069 [64] or Orlistat [65] are in early research for treating obesity, cancer and other conditions, but their effects on hypothalamic pathways controlling metabolism remain unclear. Understanding how FA blockade influences food intake and glucose homeostasis can lead to better strategies to treat metabolic syndrome.

### 3.1 Technical considerations

Constitutive Cre models may have unintended effects due to transient developmental promoter expression, impacting unexpected neurons and causing long term adaptations in metabolic and neuronal pathways [66]. For example, early embryonic POMC expression extends beyond the hypothalamus. Moreover, a subset of AgRP neurons originates from POMC precursors [39,67]. This variability can lead to inconsistent results across animal models. Tamoxifen induced Cre recombination enables precise, time specific gene deletion, bypassing developmental factors [38] and allows to test if adult deletion can replicate the phenotype observed in constitutive models. Tamoxifen, however, may impact behavior, food intake, and metabolism [6,68,69]. To minimize side effects, we found a single dose of tamoxifen IP sufficient for recombination, reducing exposure risks.

## 4 Methods

**Animals:** All experiments were conducted in male mice housed at 23 ± 1 °C with a 12 h light/dark cycle (lights on 0700–1900) fed with standard chow (Harlan, Teklad Global 16% Protein Rodent Diet 2016: 12% kcal from fat, 3 kcal/g).

To generate the FASN flox mice LoxP sites were inserted, flanking Exons 4–8 of the mouse FASN gene. Two 200 bp ssDNA UltramerTM DNA oligomers (IDT) containing a loxP site flanked by ∼80 bp homology arms were used as HDR repair templates. Guide RNAs, tracerRNA, Cas9 protein, and HDR templates were ordered from Integrated DNA Technologies (IDT). Mice were screened and validated by PCR and Sanger sequencing. Cre. Pomc-Cre mice (RRID: IMSR_JAX:005965) [37] were crossed with FASN ^flox/flox^ mice to generate mice with constitutive deletion of FASN in POMC neurons (Pomc-Cre::FASN ^flox/flox^). Recombination was verified by the presence of the delta band in tissues with high expression of POMC mRNA e.g. pituitary, but not other tissues (Figure 1 A-C). RT-qPCR of the ARH from FASN^flox/flox^ or POMC-Cre:: FASN^flox/flox^ mice confirmed FASN mRNA reduction in the POMC-Cre:: FASN^flox/flox^ mice compared to their control littermates (Figure 1 D) POMC-Cre:: FASN flox/flox mice were crossed with Ai14(RCL-tdT)-D mice (RRID: IMSR_JAX:007914) [70] mice for the electrophysiology recordings.

For adult deletion, PomcCre^ERt2^ mice (RRID: MGI:5569339) [71] were crossed with FASN ^flox/flox^ mice (POMC-Cre ^ERt2^:: FASN ^flox/flox^). For the optogenetic photo stimulation experiments, POMC-Cre ^ERt2^ mice were crossed to Ai32 mice from JAX (RRID: IMSR_JAX:012569) [72] to generate POMC-Cre:: Chr2 mice.

Mice were maintained on a C57Bl/6 J (RRID: IMSR_JAX:000664) background. Adult recombination was induced by a single dose of Tamoxifen (0.15 mg/g; Sigma–Aldrich, T5648) IP [38] suspended in corn oil (Sigma–Aldrich, C8267) administered to 10–12-weeks-old mice. Recombination was verified in Pomc-Cre ER t2:: FASN^flox/flox^ mice crossed with Ai6 (RCL-ZsGreen) (RRID:IMSR_JAX:007906) mice [70]. At the end of the experiments, mice were euthanized by IP anesthetic overdose and tissues were collected for analysis. Liver and adipose mass was normalized to total body mass.

### 4.1 Metabolic cages studies

A combined indirect calorimetry system (CaloSys Calorimetry System, TSE Systems Inc.) was used for all metabolic studies for energy balance. Body weight and age matched experimental and control animals were acclimated for 5 days in a metabolic chamber with food and water ad libitum prior to data collection. Oxygen consumption (VO2), carbon dioxide production (VCO2), respiratory exchange ratio (RER), Energy Expenditure, food intake, water intake and total locomotor activity were measured after acclimation. Data were binned using CalR Version 1.3 [73]. Food intake is expressed as kcal. For the fasting studies, mice were fasted for 24 h at ZT1 and refed with standard diet. For the glucose study, the food was removed, and dextrose (Sigma Aldrich, G8769) (1 g dextrose/kg body weight) was administered at ZT4. Food was provided again at ZT 12. For lipid administration, mice were fasted at ZT4, and 4uL/g intralipid (Sigma Aldrich, I141) gavage solution was orally administered to mice at ZT8, food was provided again at ZT 12.

### 4.2 Histology

#### 4.2.1 Double fluorescent in situ hybridization (FISH)

Animals were deeply anesthetized and perfused with PBS followed by 10% formalin, brains and pituitaries were dissected and post-fixed in 10% formalin overnight at 4 °C. The tissues were then rinsed in PBS, cryoprotected in 30% sucrose, and 30-μm thick sections were cut on a cryostat. Sections were mounted on superfrost plus microscope slides (Fisherbrand, 22-037-246) dried overnight and kept at −80C. Double FISH (RNAScope) was performed as indicated by the vendor using probes Mm-FASN-C2, 490661 and Mm-Pomc, 314081 (ACD Bio), and detected using proprietary detection reagents (RNAscope Multiplex Fluorescent V2 Detection Kit, 323110), Opal 620 (Akoya Bioscience, FP1495001KT) and Opal 690 (Akoya Bioscience, FP1497001KT) diluted 1:1000 for working solution, sections were counterstained with DAPI and coversliped with ProLong Gold Antifade Mountant (Invitrogen, P36930).

#### 4.2.2 Immunohistochemistry

Brains were obtained and cut as described above. Sections were incubated free floating with rabbit anti c-Fos primary antibody (1:1000, Abcam, 190289) overnight, rinsed 3 times with PBS and incubated for 2 h with IgG (H + L) Highly Cross-Adsorbed Donkey anti-Rabbit, Alexa Fluo Plus 594 (1:1000, Invitrogen, PIA32754) for 2 h and rinsed with PBS 3 times. Afterwards, sections were mounted and coversliped as described above. Pituitaries were cut in 25-μm thick sections, collected in slides, counterstained with DAPI and coversliped.

All slides were stored in a cold dark environment until imaging. Photomicrographs were taken using a DM6B-Z microscope (Leica) with the Leica Application Suite X software and the LSM880 Airyscan confocal microscope (Zeiss) with the ZEN-Zeiss software. Photomicrographs were managed using ImageJ 1.53c (NIH).

#### 4.2.3 Electrophysiology studies

**Slice preparation**: Brain slices were prepared from male mice (12-18 weeks-old). Briefly, male mice were anesthetized with 7% chloral hydrate via i.p. injection and transcardial perfusion was performed with a modified ice-cold artificial CSF (ACSF), in which an equiosmolar amount of sucrose was substituted for NaCl. The mice were then sacrificed, and the entire brain was removed and immediately submerged in ice-cold, carbogen-saturated (95% O2 and 5% CO2) ACSF (126 mM NaCl, 2.8 mM KCl, 1.2 mM MgCl2, 2.5 mM CaCl2, 1.25 mM NaH2PO4, 26 mM NaHCO3, and 5 mM glucose). A brain block containing the hypothalamus was made. The slices were bathed in oxygenated ACSF (32 °C–34 °C) at a flow rate of ∼2 ml/min. All electrophysiology recordings were performed at room temperature [47].

**Whole-cell recording**: The pipette solution for whole-cell recording was modified to include an intracellular dye Alexa Fluor 350 hydrazide dye (Thermofisher scientific, A10439) for whole-cell recording: 120 mM K-gluconate, 10 mM KCl, 10 mM HEPES, 5 mM EGTA, 1 mM CaCl2, 1 mM MgCl2, and 2 mM MgATP, 0.03 mM Alexa Fluor 350 hydrazide dye (pH 7.3). Epifluorescence was briefly used to target fluorescent cells, at which time the light source was switched to infrared, differential interference contrast imaging to obtain the whole-cell recording (Nikon FN-S2N Plus equipped with a fixed stage and a Andor iXon3 camera). Electrophysiological signals were recorded using an Axopatch 700 B amplifier (Molecular Devices); low pass filtered at 2–5 kHz and analyzed offline on a PC with pCLAMP programs (Molecular Devices). Membrane potential and firing rate were measured by whole-cell current clamp recordings from POMC-Cre:: TdTomato or POMC-Cre:: TdTomato:: FASN ^flox/flox^ mice in brain slices. Recording electrodes had resistances of 2.5–5 MΩ when filled with the K-gluconate internal solution. Input resistance was assessed by measuring voltage deflection at the end of the response to a hyperpolarizing rectangular current pulse step (500 ms of −10 to −50 pA).

#### 4.2.4 Metabolic tests

Metabolic tests were performed in accordance with the protocols reported by the National Mouse Metabolic Phenotyping Center. For all procedures, the basal glucose was measured before intraperitoneal injection, and additional glucose measurements were taken at 15, 30, 60, 90 and 120 min after, using a contour blood glucose monitoring system (Bayer, model 7151G).

**Glucose Tolerance Test (GTT)**: mice were fasted 6 h and given an IP injection of dextrose (Sigma Aldrich, G8769) (1 g dextrose/kg body weight). Separate cohorts of mice were used for insulin measurements.

**Insulin Tolerance Test (ITT)**:, mice were fasted 4 h and received an IP injection of insulin (0.5 UI/Kg body weight) (Humulin, Eli Lilly, R-100).

#### 4.2.5 Fasting and refeeding studies

Mice were single housed 5 days before the measurements for acclimation. Fasting experiments were performed for 24 h from 8:00 AM to 8:00 AM. Mice were refed with standard chow. Blood samples were taken at 0 and 24 h fasting, and 2 h after refeeding.

#### 4.2.6 Assessment of body composition

Percentages of fat mass and lean mass were assessed by nuclear magnetic resonance (NMR) spectroscopy using a nuclear magnetic resonance (NMR) spectroscopy (Bruker Minispec mq10 NMR 0.23T/10 MHz).

#### 4.2.7 Adrenergic blockade tests

Alpha 2 adrenergic receptor antagonist atipamezole (2 mg/kg, IP) (Sigma Aldrich, A9611) or vehicle (saline) were administered after fasting (4 h), followed by an injection of dextrose IP 30 min later (Sigma Aldrich, G8769) (1 g dextrose/kg body weight). Blood samples were taken before adrenergic blocker injection, 30 min after, before glucose administration, 15, 30 and 60 min later for glucose and insulin analysis.

#### 4.2.8 Hyperglycemic infusion

Surgery**:** Body composition was measured by NMR the day before surgery. Next, mice received a single dose of tamoxifen IP to induce recombination. The next day, mice were anesthetized with 2% isoflurane and a silicone catheter was surgically implanted in the right jugular vein, followed by flushing every other day. Body weight and general welfare was monitored daily after the implantation up to 7 days. Animals with weight loss superior to 10% were excluded from the study.

Hyperglycemic infusion: Constant hyperglycemic state was achieved by variable glucose infusion to conscious, unrestrained mice, as previously reported [74]. Briefly, a week after the jugular surgery, mice were fasted for 4–6 h prior to administration of 50% dextrose solution via the jugular catheter at a variable rate until the steady state was reached. Hyperglycemia was maintained at approx. 300 mg/dL. Steady state was achieved approx. 80 min after infusion was started and was maintained for additional 40 min. Blood samples were collected from the tail to monitor glucose. Samples taken at baseline, 10, 20, 30, 60 and 120 min during the infusion were used for insulin analysis.

#### 4.2.9 Optogenetic stimulation

Surgery: 12 to 16-week-old POMC-Cre ^ERt2^::Chr2 mice or their littermates ChR2 mice were anesthetized with 2% isoflurane and placed into a stereotaxic apparatus (David Kopf instruments). The skull was exposed via a skin incision and a small hole was drilled to hold an anchor screw. Another hole was drilled to implant a ferrule capped optic fiber aimed to the ARH AP -1.3 mm, 0.3 mm lateral and 5.85 mm ventral to the duramater. C & B Metabond Quick Self-Curing Cement System (Parkell, 553–3484) and dental acrylic to fix the ferrule capped fiber to the skull. After one week recovery, the mice were stimulated and tested (GTT). The next day, they received a dose of tamoxifen as described previously [38], followed by 1 week of washout. Afterwards, the mice were stimulated and tested (GTT).

In vivo photo stimulation: a Plexbright optogenetic stimulation system together with the Radiant software (Plexon) were used to deliver millisecond light pulses to multiple mice. A blue LED (465 nm, Plexon) was mounted on a rotary commutator and connected to a wire coated patch cables with a 200 μm diameter core, NA 0.37. The patch cables were coupled to the zirconium ferule capped fibers (1.25 OD, FZI-LC-230; Kientec Systems) implanted into the brain using a mating sleeve to deliver light to the brain. The protocol for photo stimulation consisted of 10 ms pulses, 10 pulses per second for 2 s, followed by 2 s off, repeated for 2 h. Light power exiting the fiber tip (6–8 mW) was estimated to correspond to >2.5 mW/mm^2^. Mice were connected to the optogenetic settings 4 h prior for acclimation and fasted, photo stimulation was performed for 120 min and mice brains were collected as described previously for c-fos analysis. For the metabolic test, mice were fasted for 4 h, and glucose was administered as described above, followed by immediate optogenetic stimulation.

#### 4.2.10 Hormone and metabolite analysis

Blood was collected in EDTA tubes (Sarstedt Inc 16.444.100). Plasma was isolated by centrifugation (4000 g × 10 min at 4 °C) and stored at −80 °C until analysis. Plasma leptin (Mouse/Rat Leptin ELISA, ALPCO, 22-LEPMS-E01), insulin (Mouse Ultrasensitive Insulin ELISA, ALPCO, 80-INSMSU-E01), glucagon (Mercodia Glucagon ELISA, 10-1281-01) were measured as indicated by the manufacturer. ACTH and Corticosterone were measured by RIA at the Vanderbilt Hormone Assay & Analytical Services Core.

**Analysis of gene expression by quantitative real time PCR.** Total mRNA was isolated from hypothalamus, pancreas and liver using RNA STAT-60 reagent (Tel-Test, Inc) or PureZol (Bio-Rad inc.,553–3484). For isolation of the ARH, brains were rapidly harvested and snap frozen, then placed into a chilled stainless-steel brain matrix (Electron Microscopy Sciences, 69090-C). Next, they were sectioned into coronal slices of 1 mm thickness, and the ARH region was micro dissected using a blade cut collecting only the most bottom part of the ventromedial hypothalamus. RNA concentration was assessed by UV spectroscopy using the Beer–Lambert law at 260 nm wavelength. cDNA synthesis was performed by using a High-Capacity RNA to DNA kit (Applied Biosystems, 4387406) as indicated by the company. cDNA was diluted in ultra-pure distilled water, DNase-free RNase free (Invitrogen, 10977015). mRNA transcript levels were measured in duplicate using TaqMan PCR Master Mix (Applied Biosystems, 4444557), the ABI PRISM 7900HT Sequence Detection System (Applied Biosystems, 4317596) and the CFX maestro software. Relative gene expression was calculated by the 2^−ΔΔCT^ method. Specific primers were purchased from Applied Biosystems.

#### 4.2.11 Analysis and statistics

Statistical analysis was carried out using Graph Pad Prism 9.0 software. Membrane potential values were not compensated to account for junction potential (-8 mV). Results are reported as the mean ± SEM unless indicated otherwise. The number of samples, cells or mice studied for each group is shown as individual points or noted in parentheses (n). Two group comparisons were evaluated using a 2-tailed unpaired Student's t test. In cases where experimental cohorts were matched, a paired t test was used. Groups with multiple variables were evaluated by a 2-way ANOVA. In cases where the experimental cohorts were matched, 2-way repeated measures ANOVA was used. ANOVA post-hoc analysis was performed using Sidak's multiple comparison test. Statistical significance is presented as: ^∗^(p < 0.05), ^∗∗^(p < 0.01), ^∗∗∗^(p < 0.001). Values for all data points in graphs are reported in the Supporting Data Values file.

#### 4.2.12 Study approval

All experiments were performed in accordance with the guidelines established by the Guide for the Care and Use of Laboratory Animals (National Academies Press, 2011) and were approved by The University of Texas Southwestern Medical Center Institutional Animal Care and Use Committee.

## CRediT authorship contribution statement

**Luis Leon-Mercado:** Conceptualization, Data curation, Formal analysis, Investigation, Writing – original draft, Writing – review & editing. **Yanbin Dong:** Formal analysis, Investigation, Methodology. **Bandy Chen:** Formal analysis, Investigation. **Arely Tinajero:** Investigation, Methodology. **Caleb C. Lord:** Investigation, Methodology, Resources. **Syann Lee:** Conceptualization, Formal analysis, Supervision. **Chen Liu:** Methodology, Resources. **Kevin W. Williams:** Data curation, Formal analysis, Investigation, Resources. **Joel K. Elmquist:** Conceptualization, Funding acquisition, Project administration, Resources, Supervision, Writing – original draft, Writing – review & editing.

## Declaration of competing interest

The authors declare the following financial interests/personal relationships which may be considered as potential competing interests:

Luis Leon Mercado reports financial support was provided by Leducq Foundation. Joel K. Elmquist reports financial support was provided by National Institutes of Health. Kevin W. Williams reports financial support was provided by National Institutes of Health. If there are other authors, they declare that they have no known competing financial interests or personal relationships that could have appeared to influence the work reported in this paper.

## Data Availability

Data will be made available on request.
