# Supplementary material for: Fatty Acid Synthase regulates glucose and energy homeostasis via POMC neurons and adrenergic signals
Source: Mol Metab. 2025 Jun 18;98:102177. doi: 10.1016/j.molmet.2025.102177 (PMC12268347; doi:10.1016/j.molmet.2025.102177)
Supplement: Multimedia component 1 [file mmc1.docx]

**Fatty Acid Synthase Regulates Glucose and Energy Homeostasis via POMC neurons and Adrenergic Signals**.

**Supplementary Figure 1. Validation of FASN gene deletion in POMC-Cre mice**.


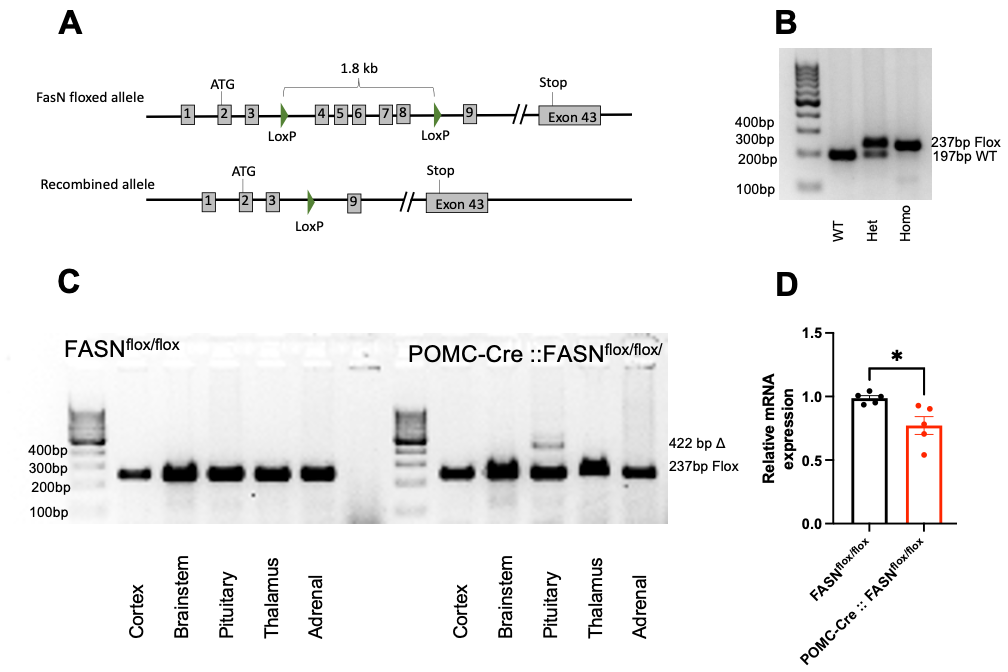


**Figure S1.**

(A) Schematic representation of the targeting strategy used for the generation of FASN floxed gene. (B) PCR analysis of the FASN floxed gene in wild type , FASN ^flox/+^ or FASN ^flox/flox^ mice.

Expected size products are 297 bp WT, 237 bp floxed alle, 422 bp for the delta band. (C) PCR of analysis of FASN recombination in cortex, brainstem, pituitary, and adrenal gland obtained from FASN^flox/flox^ mice or POMC-Cre :: FASN^flox/flox^ mice. (D) RT- qPCR analysis shows a reduction of FASN mRNA expression in the ARH of POMC-Cre :: FASN^flox/flox^ mice compared to FASN^flox/flox^ mice (Unpaired t test, t=2.950, df=8, P=0.0184) (n=5,5). Data are expressed as mean ± SEM. Significance is noted as *p<0.05, **p<0.01, ***p<0.001.

**Supplementary figure 2. RER and locomotor activity are comparable to control mice in FASN POMC deficient mice.**

**
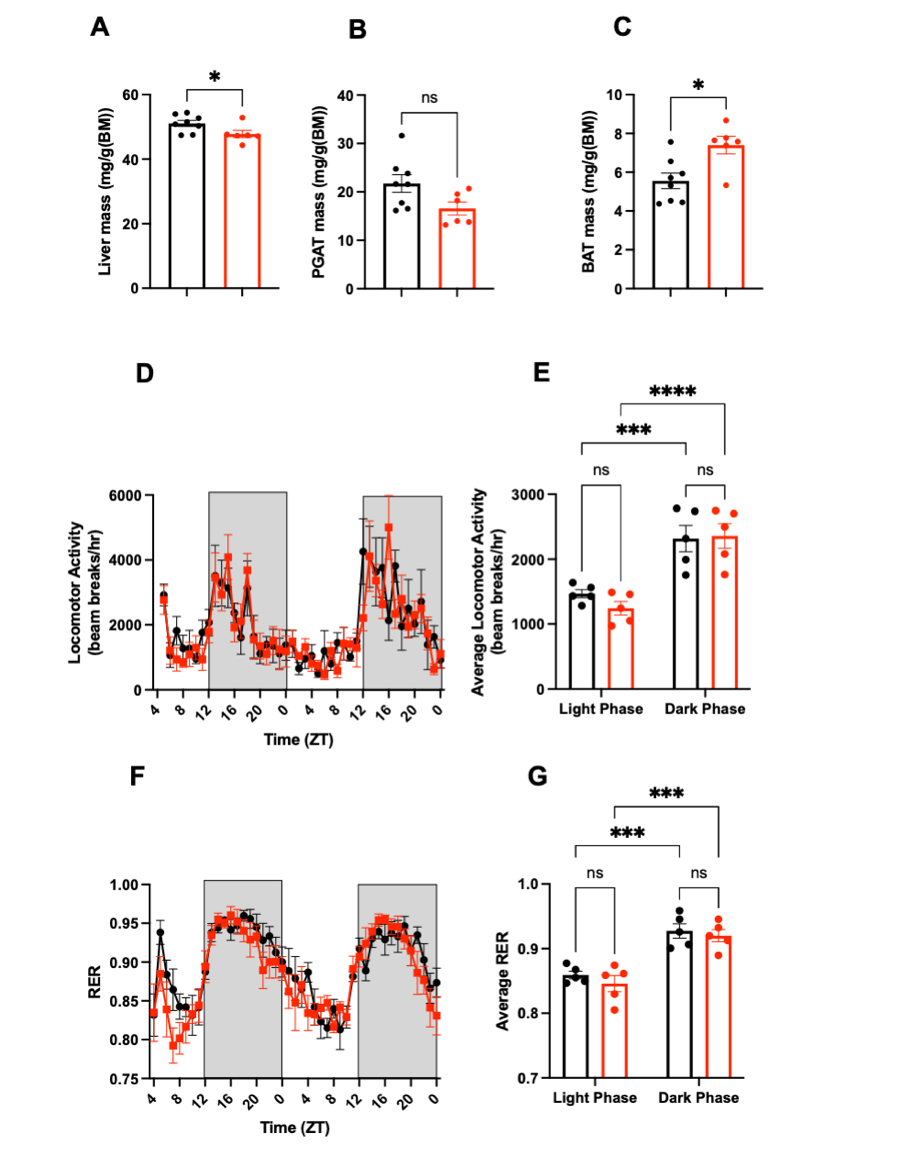
**

**Figure S2.**

(A-C) Organ mass relative to total body mass at 16 weeks old. (A) Liver (Time x Genotype F (1, 24)=0.6735 P=0.4199, Time F (1, 24)=0.01375 P=0.9076, Genotype F (1, 24)=4.514 P=0.0441) (n=8, 6), (B) PGAT (Time x Genotype F (1, 24)=1.416 P=0.2457, Time F (1, 24)=0.03967 P=0.8438, Genotype F (1, 24)=2.555 P=0.1231) (n=8, 6) and (C) BAT (Time x Genotype F (1, 24)=0.9906 P=0.3295, Time F (1, 24)=0.02022 P=0.8881, Genotype F (1, 24)=7.769 P=0.0102) (n=8, 6). (D, E)Analysis of locomotor activity by metabolic chambers across 24h. (D) Comparison of the locomotor activity pattern across 24h (Time x Genotype F (43, 344)=1.060 P=0.3767, Time F (5.459, 43.67)=7.232 P<0.0001). (E) Average locomotor activity in the light phase (ZT 0 - ZT 12) and dark phase (ZT 12 – ZT 24) (Time x Genotype F (1, 8)=1.739 P=0.2238, Time F (1, 8)=93.15 P<0.0001) (n=5, 5). (F, G)Analysis of RER by metabolic chambers across 24h. (F) Comparison of the RER pattern across 24h (Time x Genotype F (44, 352)=1.153 P=0.2418, Time F (44.00, 352.0)=15.19 P<0.0001). (G) Average RER in the light phase (ZT 0 - ZT 12) and dark phase (ZT 12 – ZT 24) (Time x Genotype F (1, 8)=0.1397 P=0.7183, Time F (1, 8)=77.51 P<0.0001) (n=5, 5).Data are expressed as mean ± SEM. RM two-way ANAOVA. Significance is noted as *p<0.05, **p<0.01, ***p<0.001.

**Supplementary Figure 3. Histological analysis of the pituitary gland**.


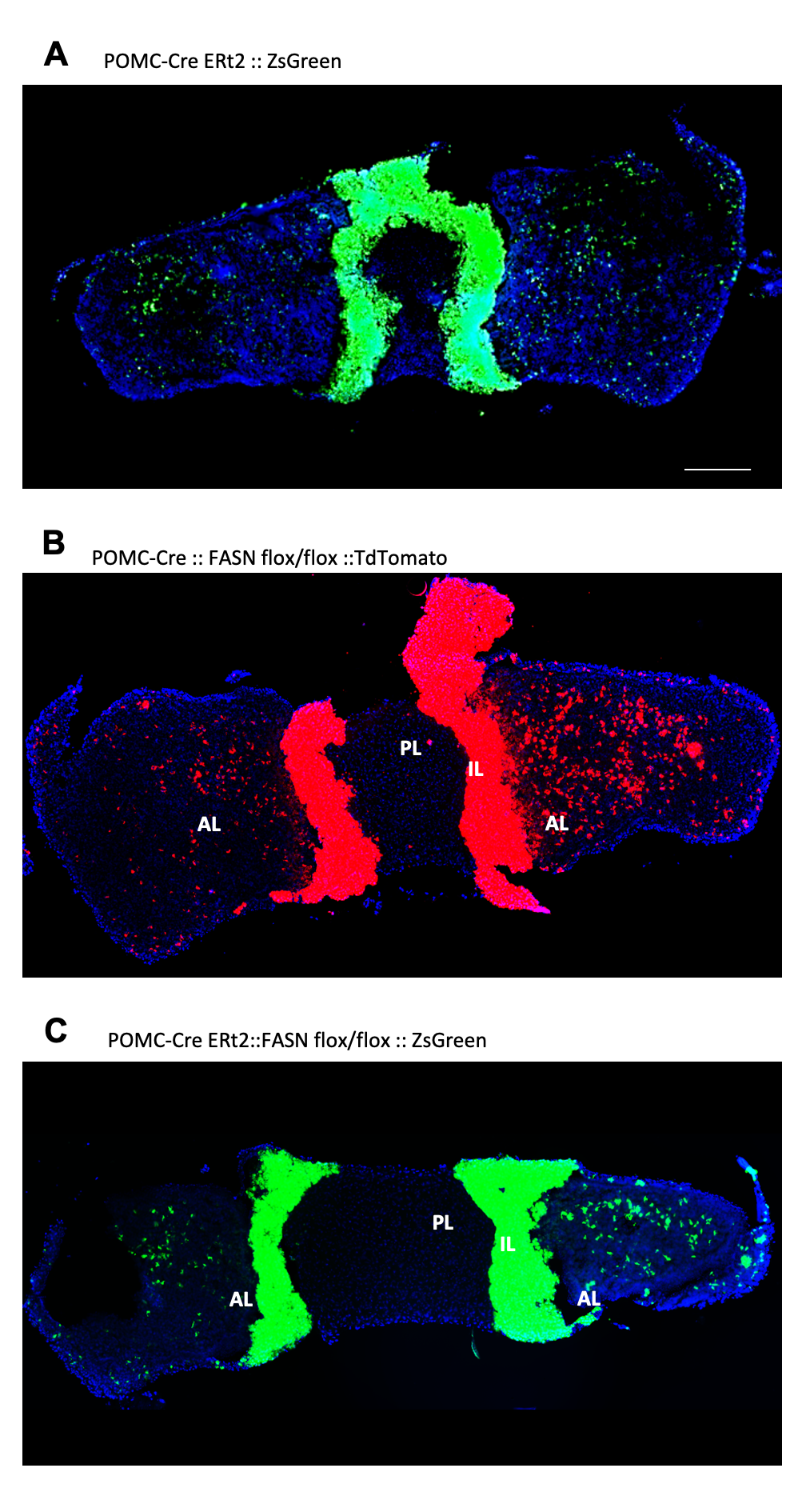


**Figure S3**.

(A) ZsGreen expression after tamoxifen administration to POMC-Cre ^ERt2^ :: ZsGreen mice.

(B) TdTomato expression in the pituitary of POMC-Cre :: FASN^flox/flox^ :: TdTomato mice.

(C) ZsGreen expression after tamoxifen administration to POMC-Cre ^ERt2^ :: FASN^flox/flox^ ZsGreen mice. TdTomato (red), ZsGreen (green), DAPI (blue). Anterior lobe (AL), Intermediate Lobe (IL), Posterior Lobe (PL). Scale bar 250 um.

**Supplementary figure 4. Locomotor activity and RER are comparable to control mice in FASN POMC deficient mice after fasting and refeeding.**

**
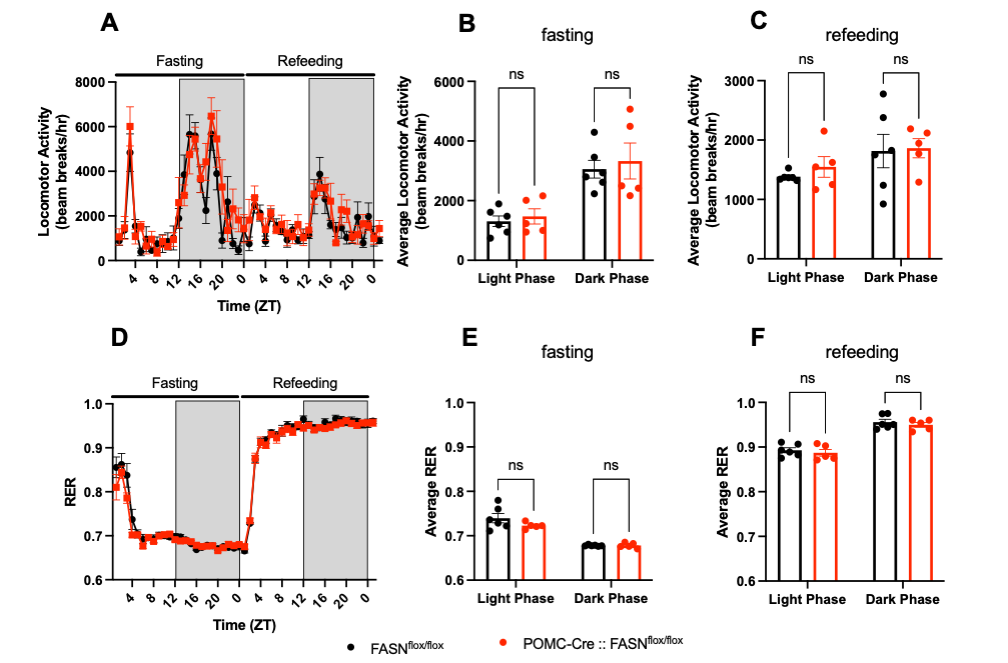
Figure S4.**

(A-C) Analysis of locomotor activity by metabolic chambers during 24h fasting followed by 24h refeeding. (A) Comparison of the locomotor activity pattern during 24h fasting (Time x Genotype F (24, 216)=1.394, P=0.1114 Time F (4.635, 41.71)=19.49 P<0.0001) and 24h refeeding (Time x Genotype F (24, 216)=0.8863 P=0.6208, Time F (4.954, 44.59)=6.685 P=0.0001). (B) Average locomotor activity during the light and dark phase of fasting (Time x Genotype F (1, 9)=0.06172 P=0.8094, Time F (1, 9)=64.53 P<0.0001), and (C) refeeding (Time x Genotype F (1, 9)=0.1387 P=0.7182, Time F (1, 9)=5.463 P=0.0442) (n=6, 5). (D-F) Analysis of RER by metabolic chambers during 24h fasting followed by 24h refeeding. (D) Comparison of the RER pattern during 24h fasting (Time x Genotype F (23, 207)=2.023 P=0.0052, Time F (3.000, 27.00)=54.64 P<0.0001) and 24h refeeding (Time x Genotype F (24, 216)=0.6974 P=0.8516, Time F (5.466, 49.20)=326.9 P<0.0001). (E) Average RER during the light and dark phase of fasting (Time x Genotype F (1, 9)=2.088 P=0.1824, Time F (1, 9)=83.91 P<0.0001), and (F) refeeding (Time x Genotype F (1, 9)=0.00677 P=0.9362, Time F (1, 9)=828.6 P<0.0001) (n=6, 5). Data are expressed as mean ± SEM. RM two-way ANAOVA. Significance is noted as *p<0.05, **p<0.01, ***p<0.001.

**Supplementary figure 5.** **Validation of FASN gene deletion in POMC-Cre^ERt2^:: FASN^flox/flox^ mice**.

**Figure S5.**

Visualization of POMC mRNA and FASN mRNA in the ARH by Fluorescent in situ hybridization. (A-K) Recombination decreases co-expression of FASN and POMC mRNA in the ARH of POMC-Cre ^Ert2^ :: FASN^flox/flox^ mice. (A) Low magnification fluorescence photomicrographs of POMC and FASN mRNA in the ARH. (B) FASN mRNA (red) (C) POMC mRNA (green) (D) Higher magnification of the area delineated in (A), illustrating the presence of POMC and FASN mRNA in the same neurons (withe arrows).(E-K) Absence of co-expression of FASN and POMC mRNAs in the ARH of POMC-Cre ^Ert2^ ::FASN^flox/flox^ mice. (E) Low magnification fluorescence microphotographs of POMC and FASN mRNA in the ARH.(F) FASN mRNA (red), (G) POMC mRNA (green), (H) Higher magnification of the area delineated in (E), illustrating minimal colocalization of POMC and FASN mRNA in the ARH of POMC-Cre^ERt2^:: FASN^flox/flox^ mice. (I) FASN mRNA (red), (J) ZsGreen (green) and (K) merge illustrating minimal colocalization of FASN mRNA and Cre dependent fluorescent reporter after tamoxifen-induced recombination. Quantification of co-expression of FASN mRNA and POMC mRNA in the ARH of POMC-Cre Ert2 :: FASN^flox/flox^ mice (L) before (t=2.833, df=4, P= 0.0472) and (M) after recombination (t=9.979, df=4, P = 0.0006) (L) and co-expression of FASN mRNA and ZsGreen after recombination (t=3.923, df=4, P = 0.0172). 3V, third ventricle: ARH Arcuate Nucleus, ME, Median Eminence; VMH, Ventromedial Hypothalamus. Scale bar 100 um (A, E) 50 um (D, H). Data are expressed as mean ± SEM. Unpaired t test. Significance is noted as *p<0.05, **p<0.01, ***p<0.001

**Supplementary figure 6**. **Determination of gene expression in the arcuate nucleus of the hypothalamus and liver of FASN^flox/flox^ and POMC-Cre^ERt2^:: FASN^flox/flox^ after tamoxifen administration.**


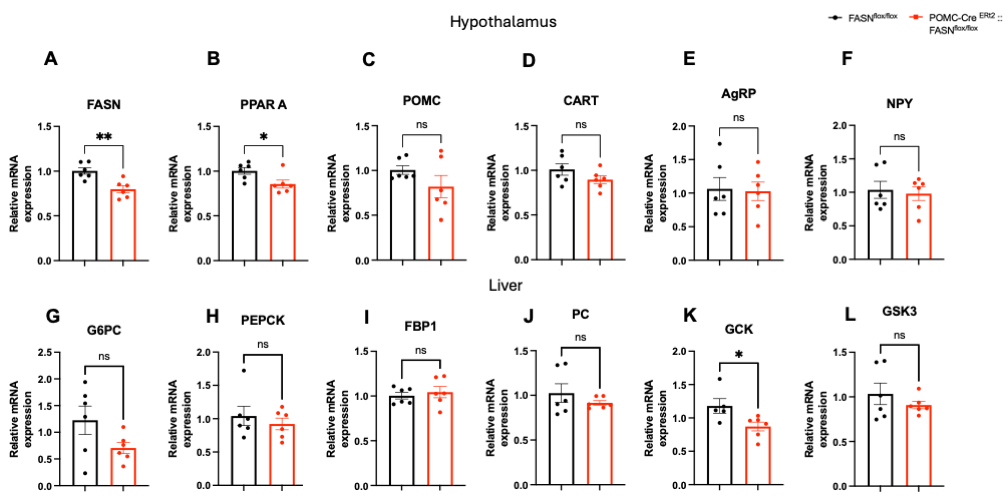


**Figure S6**.

(A-F) Relative mRNA levels in the arcuate nucleus of the hypothalamus (A) FASN (t=3.783, df=10 P=0.0036), (B) PPARa (t=2.514, df=10 P=0.0307), (C) POMC (t=1.391, df=10 P=0.1944), (D) CART (t=1.483, df=10 P=0.1690), (E) AgRP (t=0.1611, df=10 P=0.8752) and

(F) NPY (t=0.3518, df=10 P=0.7323) (n=6, 6).(G-L) Relative mRNA levels in the liver (G) G6PC (t=1.819, df=10, P=0.099) (H) PEPCK (t=0.7077, df=10, P=0.4953) (I) FBP1 (test t=0.5505, df=10, P=0.5941) (J) PC (df=10, P=0.3321, P=0.3321), (K) GCK (t=2.357, df=10, P=0.0402), (L) GSK3 (t=0.9857, df=10, P=0.3475). Samples were collected from ZT 0 to ZT 2.Values were calculated by the 2^-ΔΔCT^ method. Data are expressed as mean ± SEM. Unpaired t test. Significance is noted as *p<0.05, **p<0.01, ***p<0.001

**Supplementary figure 7. Adult deletion of FASN in POMC decreases perigonadal and perirenal fat mass.**


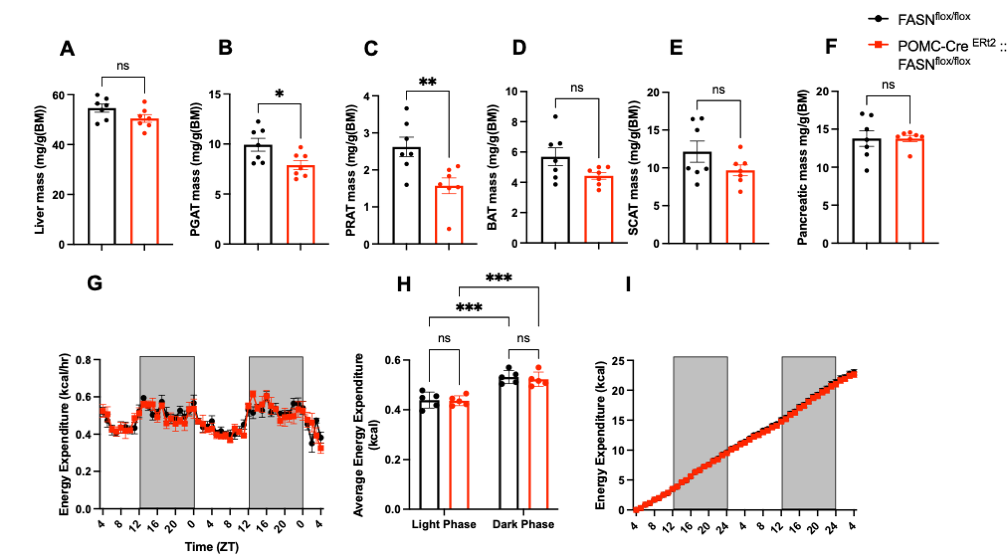


**Figure S7.** (A-F) Organ mass relative to total body mass after tamoxifen induced recombination. (A) Liver (t=1.840, df=12, P=0.0907), (B) PGAT (t=2.578, df=12, P=0.0242), (C) PRAT (t=3.126, df=12, P=0.0088), (D) BAT (t=2.008, df=12, P=0.0677), (E) SCAT (t=1.578, df=12, P=0.1406), (F) Pancreatic mass (t=0.002352, df=12, P=0.9982)(n=7, 7). (G-I) Analysis of Energy Expenditure by metabolic chambers. (G) Comparison of the Energy Expenditure pattern (Time x Genotype F (48, 384)=0.9592, P=0.5536, Time F (6.574, 52.59)=9.622 P<0.0001). (H) Average Energy Expenditure in the light phase (ZT 0 - ZT 12) and dark phase (ZT 12 – ZT 24) (Time x Genotype F (1, 8)=0.1301 P=0.7277, Time F (1, 8)=90.20 P<0.0001).(I) Comparison of the cumulative Energy Expenditure (Time x Genotype F (48, 384)=0.4279 P=0.9997, Time F (1.208, 9.663)=4610 P<0.0001) (n=5, 5). Data are expressed as mean ± SEM. Unpaired t test, RM two-way ANOVA, Significance is noted as *p<0.05, **p<0.01, ***p<0.001**.**

**Supplementary figure 8. RER and locomotor activity are comparable to control mice after adult FASN deletion in POMC during fasting and refeeding.**

**
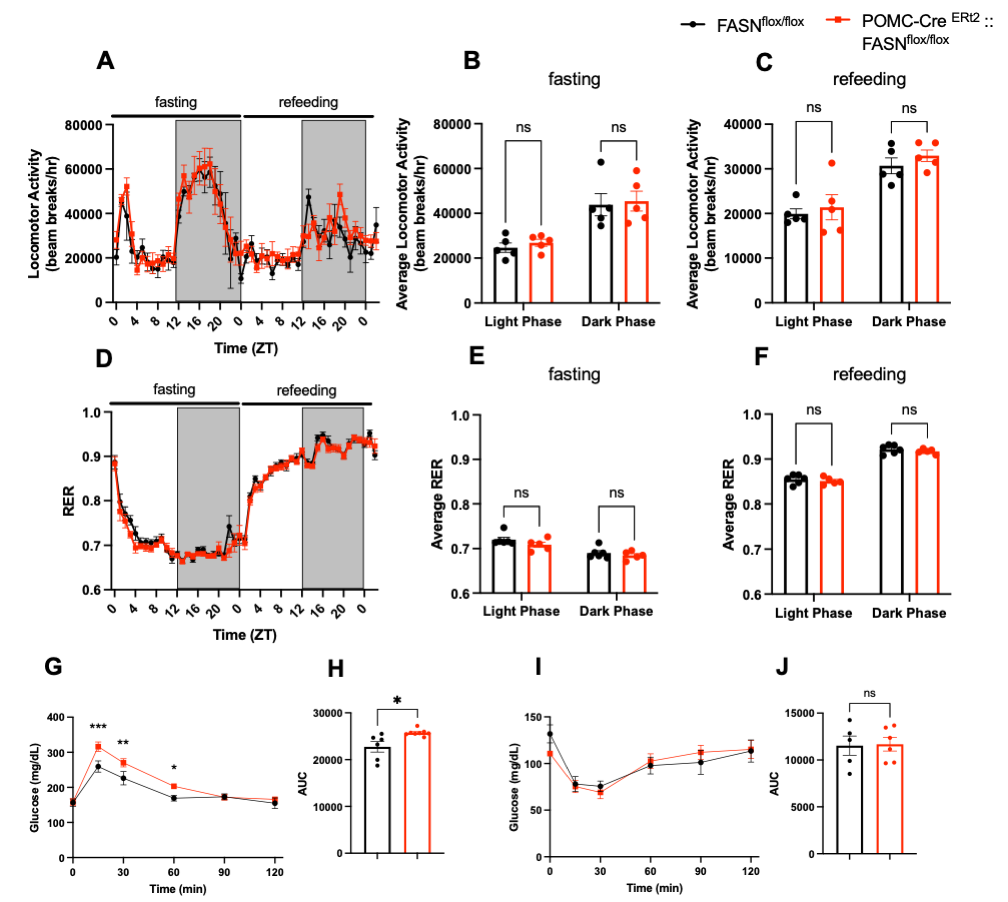
**

**Figure S8.**

(A-C) Analysis of locomotor activity by metabolic chambers during 24h fasting followed by 24h refeeding.(A) Comparison of the locomotor activity pattern during 24h fasting (Time x Genotype F (24, 192)=0.4993 P=0.9766, Time F (3.910, 31.28)=17.74 P<0.0001) and 24h refeeding (Time x Genotype F (23, 184)=1.422 P=0.1047 Time F (5.298, 42.39)=6.151 P=0.0002). (B) Average locomotor activity during the light and dark phase of fasting (Time x Genotype F (1, 8)=0.01013 P=0.9223, Time F (1, 8)=40.29 P=0.0002), and (C) refeeding (Time x Genotype F (1, 8)=0.03874 P=0.8489 Time, F (1, 8)=31.05 P=0.0005) (n- 6, 5). (D-F) Analysis of RER by metabolic chambers during 24h fasting followed by 24h refeeding. (D) Comparison of the RER pattern during 24h fasting (Time x Genotype F (24, 216)=1.024 P=0.4372 Time, F (4.023, 36.20)=39.31 P<0.0001) and 24h refeeding (Time x Genotype F (23, 207)=0.6788 P=0.8636, Time F (6.236, 56.12)=106.4 P<0.0001). (E) Average RER during the light and dark phase of fasting (Time x Genotype F (1, 9)=0.2299 P=0.6430, Time F (1, 9)=19.48 P=0.0017), and

(F) refeeding (Time x Genotype F (1, 9)=0.05768 P=0.8156, Time F (1, 9)=1053 P<0.0001) (n=6, 5). (G-J) Glucose and insulin tolerance tests. (G) Blood glucose (Time x Genotype F (5, 60)=2.506 P=0.0398, Time F (5, 60)=50.51 P<0.0001)and (H) AUC values after glucose administration (t=3.010, df=12 P=0.0109)(n=6, 8). (I) blood glucose (Time x Genotype F (5, 45)=2.210 P=0.0697, Time F (3.346, 30.11)=28.93 P<0.0001) and (J) AUC values after insulin administration (t=0.1176, df=9 P=0.5774) (n=5, 6). Data are expressed as mean ± SEM. RM two-way ANOVA, Unpaired t test, Significance is noted as *p<0.05, **p<0.01, ***p<0.001**.**
